# Fatty acid-binding protein 4 drives microglia-mediated neuroinflammation through promoting S100A9 expression and lipid droplet accumulation after intracerebral hemorrhage

**DOI:** 10.1186/s12974-025-03573-6

**Published:** 2025-11-07

**Authors:** Jiaqing He, Zijuan Qin, Haixiao Liu, Yaning Cai, Hao Wu, Tinghao Wang, Qing Hu, Yanni Xu, Pan Yang, Xun Wu, Yan Qu, Wei Guo

**Affiliations:** 1 Department of Neurosurgery, Xi’an Medical University, Xi’an, Shaanxi 710021 China; 2 Department of Neurosurgery, Tangdu Hospital, the Fourth Military Medical University, Xi’an, Shaanxi 710032 China; 3 School of Medicine, Northwest University, 229 Taibai North Road, Xi’an, 710069 China; 4 Department of Biomedical Engineering, Fourth Military Medical University, Xi’an, Shaanxi 710032 China; 5 Department of Orthopedics, Tangdu Hospital, the Fourth Military Medical University, Xi’an, Shaanxi 710032 China; 6 Department of Neurosurgery, The 83rd Affiliated Hospital of Xinxiang Medical University, Xinxiang, Henan China; 7 Department of Neurosurgery, First Affiliated Hospital of Gannan Medical University, Ganzhou City, 341000 China; 8 Department of Emergency, Tangdu Hospital, Fourth Military Medical University, Xi’an, Shaanxi 710032 China

**Keywords:** Intracerebral hemorrhage, FABP4, Microglia, Neutrophil, Lipid droplet, S100A9

## Abstract

**Background:**

As primary immune sentinels of the central nervous system (CNS), microglia respond rapidly to acute brain injury and engage in dynamic crosstalk with infiltrating peripheral immune cells. This interplay critically shapes the neuroinflammatory microenvironment—a key determinant of secondary brain injury (SBI) following intracerebral hemorrhage (ICH). Fatty acid-binding protein 4 (FABP4), an adipokine associated with metabolic disorders, is recognized as a pivotal modulator of inflammatory responses; however, its role in ICH-induced SBI remains undefined.

**Objectives:**

To investigate the pathogenic functions of FABP4 in microglia after ICH, elucidate its molecular mechanisms, and develop targeted therapeutic strategies.

**Methods:**

Blood and brain tissue samples from ICH patients were analyzed to evaluate the relationships between FABP4 expression and prognosis. Behavioral tests, Nissl staining, and Golgi-Cox staining were used to quantify neuronal damage. Immunofluorescence and flow cytometry were used to assess microglial activation and immune cell infiltration. Transcriptomic, proteomic, co-immunoprecipitation, western blotting, and ChIP‒qPCR analyses were used to examine the FABP4 regulatory network. Brain-targeted nanoparticles were engineered to deliver FABP4-specific siRNA.

**Results:**

Clinical analyses revealed microglia-specific FABP4 upregulation in ICH patients, correlating with poor neurological outcomes. Microglial Fabp4 knockout in mice attenuated neuronal loss, ameliorated cerebral edema, and enhanced functional recovery after ICH. Mechanistically, FABP4 promoted lipid droplet accumulation and inhibited the ubiquitin–proteasome-mediated degradation of S100A9 in microglia, synergistically amplifying neuroinflammation. Moreover, the activity of FABP4 in microglia facilitated neutrophil transendothelial migration into the brain parenchyma, exacerbating injury via the release of neutrophil extracellular traps (NETs). Finally, pharmacological FABP4 inhibition using brain-targeted nanoparticles conferred significant neuroprotective effects in ICH models.

**Conclusion:**

This study establishes that FABP4 acts as a novel orchestrator of post-ICH neuroinflammation through dual enzymatic and nonenzymatic pathways. We also demonstrate a targeted nanotherapeutic strategy to suppress FABP4 and improve neurological outcomes.

**Supplementary Information:**

The online version contains supplementary material available at 10.1186/s12974-025-03573-6.

## Introduction

Intracerebral hemorrhage (ICH) represents 10–15% of all stroke cases and has the highest mortality and morbidity rates among stroke subtypes [1–3]. After the initial hemorrhage, inflammatory and cytotoxic responses to the hematoma and its breakdown components cause sustained secondary brain injury to the surrounding parenchyma [4]. However, the precise regulatory mechanisms underlying this process remain elusive.

As specialized macrophages that reside in the central nervous system (CNS), microglia serve as the primary immune defense barrier within the brain and spinal cord [5]. Activated microglia emerge as principal producers of cytokines, chemokines, proteases, and immunomodulatory factors within the CNS [6]. Crucially, their interplay with peripheral immune components (neutrophils, monocytes, macrophages, T cells, and NK cells) shapes the cerebral inflammatory milieu [7]. These cellular dynamics are strongly correlated with cytotoxic processes and neuronal loss in proinflammatory environments, underscoring the need to investigate the underlying mechanism of microglial activation and intercellular crosstalk [8, 9].

Fatty acid-binding protein-4 (FABP4) is an adipokine that governs fatty acid trafficking and metabolic signaling, playing a crucial role in metabolic dysfunction because of its proinflammatory effects [10, 11]. Clinical studies have implicated FABP4 in diverse pathologies [12–15], including cardiovascular diseases [16], hypertension [17], obesity [18], diabetes [19], and insulin resistance [20]. Growing evidence has shown its involvement in CNS disorders such as ischemic stroke [21], glioma, and metabolic dementia. Elevated serum FABP4 levels in ischemic stroke patients correlates with early mortality and unfavorable outcomes [22]. However, its pathophysiological role in ICH remains undefined. Given its established role as a pivotal modulator of inflammatory and metabolic processes in peripheral diseases, and its recent implication in ischemic stroke outcomes, we hypothesized that FABP4 might also play a critical role in mediating microglial dysfunction and neuroinflammation after ICH.

This study aimed to elucidate the mechanisms underlying the pathogenic activities of microglia after ICH. Clinical studies revealed a significant positive correlation between elevated serum FABP4 levels and unfavorable neurological outcomes in ICH patients. Experimental investigations demonstrated that FABP4 expression was increased after ICH and was mainly localized to microglia in the brain in both patients and mice. Furthermore, increased FABP4 expression promoted lipid droplet accumulation and impaired S100A9 ubiquitination. These molecular events triggered pro-inflammatory microglial activation, leading to the release of neurotoxic factors and facilitating peripheral neutrophil transendothelial migration into the brain parenchyma; both genetic ablation and pharmacological inhibition of FABP4 resulted in attenuated neuroinflammation, reduced neuronal loss, and improved functional recovery after ICH. Collectively, our findings suggest a novel role for FABP4 in orchestrating brain inflammation after ICH and offer a potential therapeutic strategy for microglia-mediated neuroinflammation.

## Results

### FABP4 expression is associated with perihematomal edema volume and neurological outcomes in ICH patients

**Fig. 1:**
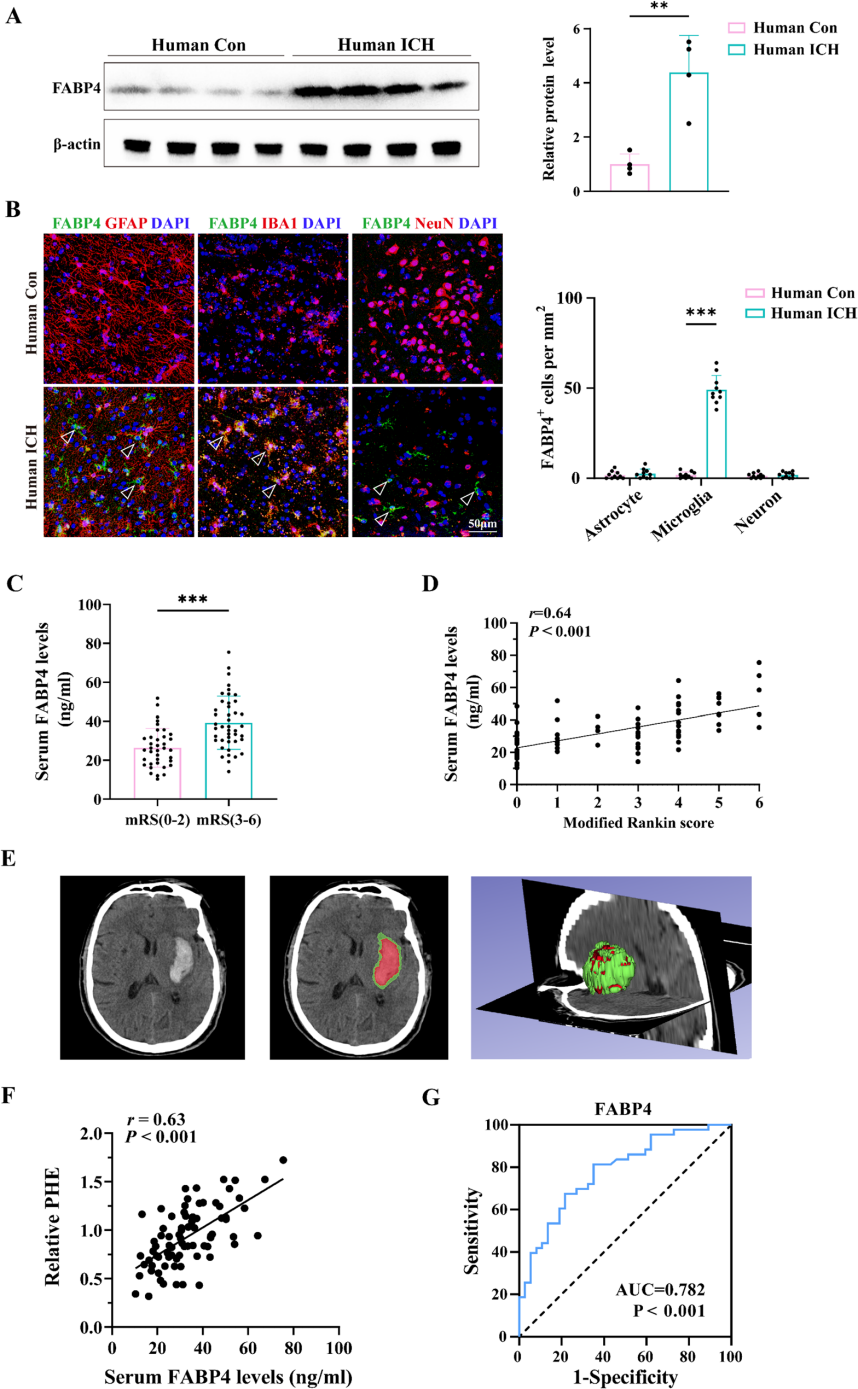
FABP4 expression was linked to perihematomal edema and neurological outcomes in ICH patients.** A **Western blot analysis of FABP4 expression levels in brain tissue samples from ICH patients and controls (4 samples for ICH group, 4 samples for control group, Student’s t-test). **B** Representative immunostaining images and statistical analysis of FABP4 expression in astrocytes (GFAP⁺ cells), microglia (IBA1⁺ cells), and neurons (NeuN⁺ cells) in brain tissue from ICH patients and controls. Scale bar: 50 μm. *n* = 10 per group, Student’s t-test. **C** Serum FABP4 levels were measured in patients on day 1 after ICH There were 37 patients in the favorable group (mRS = 0–2) and 43 in the unfavorable group (mRS = 3–6). **D** The relationship between FABP4 and the modified Rankin score at six months after ICH is shown by the Pearson correlation coefficient (r) and P value (P) (*r* = 0.64, *P* < 0.001). **E** The CT scan and 3D model reveal a hematoma and the perihematomal area on the right side. The brain hemisphere is depicted with red indicating the hematoma and green representing the edema. **F** The Pearson correlation coefficient (r) and P value (P) are calculated to determine the association between serum FABP4 and relative PHE (*r* = 0.63, *P* < 0.001). *n* = 80 in ICH patient group. **G** ROC curve for individual serum FABP4 expression on day 1 to separate favorable (mRS = 0–2) outcomes from unfavorable (mRS = 3–6) outcomes 6 months later (AUC = 0.782). Data are expressed as mean ± SD. *p < 0.05; **p < 0.01; ***p < 0.001

To investigate the potential role of FABP4 in secondary brain injury after ICH, we initially evaluated FABP4 levels in surgically resected brain contusion tissues from four ICH patients. Histologically distinct brain samples obtained from four non-ICH patients who underwent deep tumor resection served as the comparison group. Western blot analysis revealed markedly higher FABP4 levels in ICH-affected brain tissue samples than in control tissue samples (Fig. 1A). Immunostaining revealed that FABP4 was predominantly expressed in microglia but was also expressed at relatively low levels in astrocytes and neurons after ICH (Fig. 1B). Furthermore, to definitively confirm the microglial identity of FABP4-expressing cells, we performed co-staining with TMEM119, a specific microglial marker [23]. This revealed that the majority of FABP4 + cells were TMEM119+, identifying them as resident microglia rather than infiltrating macrophages (Figure S1A-B, Supporting Information).

To investigate the correlation between elevated FABP4 levels and prognosis, we subsequently collected serum samples from 80 ICH patients, measured the FABP4 levels in these samples, and assessed the 6-month modified Rankin scale (mRS) scores. Multivariate logistic regression indicated that elevated serum FABP4 served as an independent risk factor for unfavorable neurological outcomes (Table 1; Fig. 1C). Pearson’s correlation analysis revealed a positive correlation between serum FABP4 levels and 6-month mRS scores (*r* = 0.64, *P* < 0.001; Fig. 1D). Given the established role of the perihematomal edema (PHE) volume as a marker of secondary brain injury after ICH, we measured the PHE volume using 3D Slicer software (Fig. 1E). Serum FABP4 levels were positively correlated with the PHE volume, as demonstrated by Pearson’s correlation coefficient (r) (*r* = 0.63, *P* < 0.001; Fig. 1F). Receiver operating characteristic (ROC) analysis indicated a plasma FABP4 concentration cutoff value of 29.01 ng/mL, with 81.40% sensitivity and 64.86% specificity for predicting unfavorable outcomes (AUC = 0.782; Fig. 1G). Overall, the levels of FABP4 were found to be associated with perihematomal edema and neurological outcomes in ICH patients.

**Table 1:** Characteristics of patients with ICH and multivariate logistic regression analysis

Characteristic	Favorable outcome [*n* = 37]	Unfavorable outcome [*n* = 43]	*p*-value	Odds ratio (OR) [95%CI]	*p*-value
Age	58.46 ± 9.671	63.2093 ± 9.316	0.033	0.962(0.875,1.057)	0.420
(Mean ± SD)					
Sex			0.202		
Female	12(32.4%)	20(46.5%)			
Male	25(67.6%)	23(53.5%)			
Glasgow Coma Scale (GCS)			<0.001	1.606(1.085,2.377)	0.018
≥ 12	28(75.7%)	10(23.3%)			
<12	9(24.3%)	33(76.7%)			
Smoking	18(48.6%)	22(52.2%)	0.823		
Diabetes	3(8.1%)	5(11.6%)	0.603		
White blood cell (IQ range)	8.9538(6.4700,10.4250)	8.6737(6.9600,9.8700)	0.690		
Craniocerebral disease	4(10.8%)	11(25.6%)	0.100		
PHE vol, ml	8.6989 ± 4.06351	18.5007 ± 8.12515	<0.001	0.826(0.702,0.972)	0.022
(Mean ± SD)					
FABP4, ng/ml	26.3635 ± 9.93049	39.2258 ± 13.62244	<0.001	0.915(0.839,0.997)	0.042
(Mean ± SD)					
Hematoma vol, ml	18.1721 ± 5.87481	29.0190 ± 9.59151	<0.001	0.881(0.786,0.987)	0.029
(Mean ± SD)					
Hypertension			0.059		
No	14(37.8%)	8(18.6%)			
Yes	23(62.2%)	35(81.4%)			

### ICH induces the transcriptional upregulation of FABP4 in mouse microglia

**Fig. 2:**
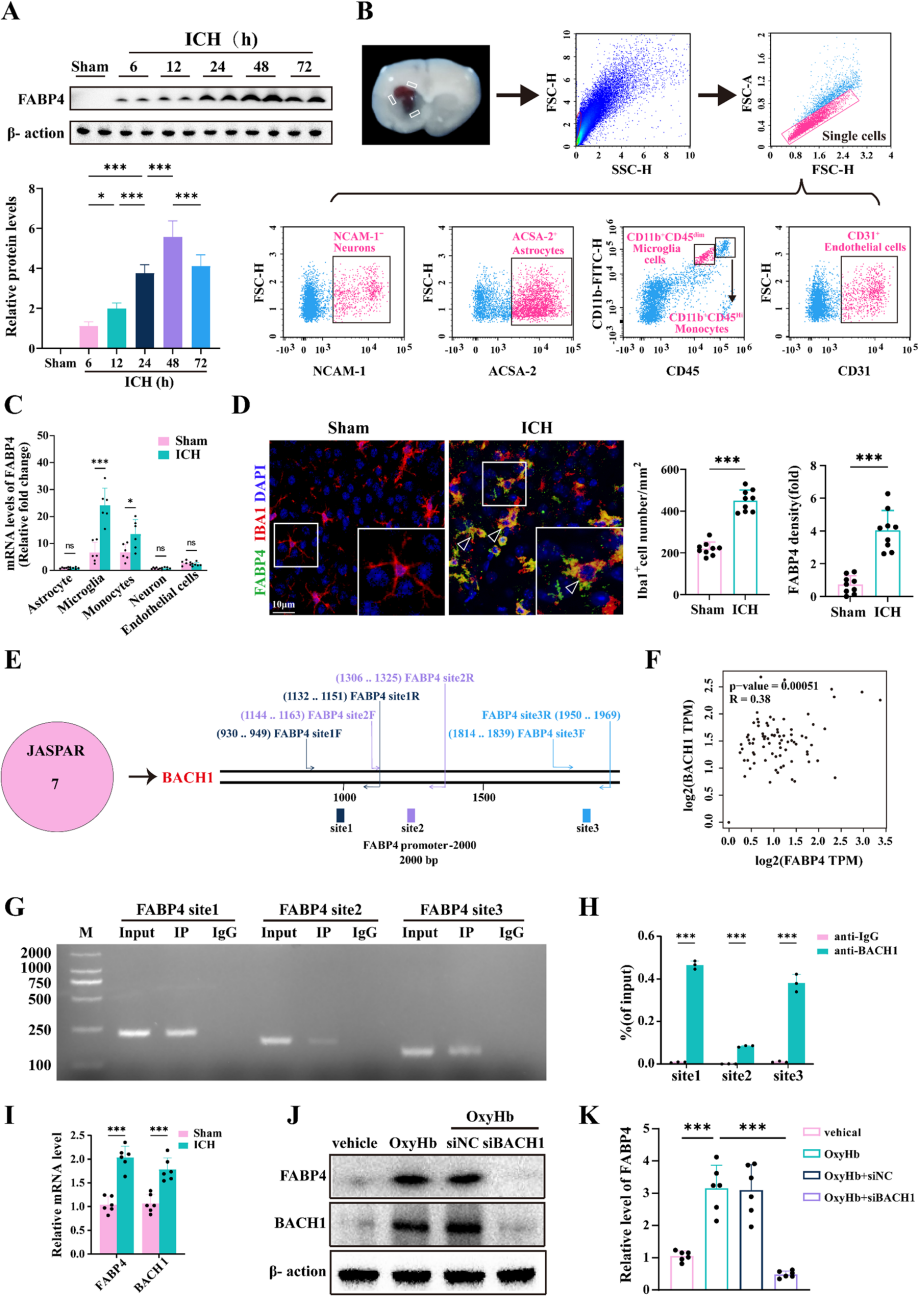
FABP4 upregulation was observed in mice microglia after ICH. **A** Representative immunoblots for FABP4 of the perihematomal brain tissues at various time points after ICH and the same region of the mice 24 h after sham operation (*n* = 6 per group, one-way ANOVA). **B**-**C** Schematic of neurons, astrocytes, microglia, monocytes, and endothelial cells isolation from ICH mice. Relative expression of FABP4 in the sorted cells as determined by qPCR (*n* = 6 per group, Student’s t-test). **D** Representative images of immunofluorescence and statistical analysis of co-staining of FABP4 with Iba1 in mice brain Sect. 48 h after ICH. Scale bar: 10 μm. Data are represented as the number of Iba1 cells per mm^2^ and the quantification analysis of the relative fluorescence intensity in FABP4 (*n* = 9 per group, Student’s t-test). **E** The schematic diagram of the binding site sequences where the transcription factors screened from the database interact with the FABP4 promoter. **F** Correlation between the FABP4 and BACH1 based on a the the brain putamen (basal ganglia) tissue of the Genotype-Tissue Expression (GTEx) in the GEPIA database. **G** ChIP analysis was performed to assess BACH1’s binding to the FABP4 promoter. **H** q-PCR analysis was carried out on DNA obtained from ChIP (*n* = 3 per group, one-way ANOVA). **I** PCR assay of the mRNA level of FABP4 and BACH1 after ICH (*n* = 6 per group, Student’s t-test). **J**-**K** Western blot analysis was conducted to examine the expression levels of FABP4 and BACH1 in primary microglia under various treatments, along with the corresponding statistical evaluation (*n* = 6 per group, Student’s t-test). Data are presented as means ± SD. *p < 0.05, **p < 0.01, ***p < 0.001

The dynamic expression of FABP4 was then assessed after ICH in mice. Notably, both the mRNA and protein levels of FABP4 were significantly increased in the brain at 24 h post-ICH, with a peak at 48 h and a sustained increase for at least 72 h (Fig. 2A). Using flow cytometry, we demonstrated that FABP4 levels were markedly increased in microglia with only a modest increase in macrophages, but did not significantly change in neurons, astrocytes, or endothelial cells (Fig. 2B-C). Immunofluorescence costaining and colocalization analyses also revealed that FABP4 was colocalized with IBA1 in the perihematomal area (Fig. 2D). In line with clinical observations in patients, mouse experiments demonstrated that ICH induces the specific upregulation of FABP4 expression in microglia.

The mechanisms responsible for the upregulation of FABP4 were subsequently explored. Following ICH, both the mRNA and protein levels of FABP4 were elevated, indicating that FABP4 expression is regulated at the transcriptional level. The JASPAR database was used to further analyze the potential transcription factors for FABP4. The top candidate identified was BACH1, which was selected for further validation (Fig. 2E). Genotype-Tissue Expression (GTEx) in the GEPIA database was used to examine the relationship between the expression of FABP4 and BACH1 in the brain putamen (basal ganglia) tissue [24]. Pearson correlation analysis revealed a moderate positive correlation between BACH1 and FABP4 expression (Fig. 2F). Additionally, chromatin immunoprecipitation (ChIP) combined with quantitative polymerase chain reaction (qPCR) verified the direct attachment of BACH-1 to the FABP4 promoter region (Fig. 2G-H). PCR analysis revealed that BACH1 expression was also upregulated in mice after ICH (Fig. 2I). The siRNA-mediated silencing of BACH-1 expression significantly inhibited the OxyHb-induced upregulation of FABP4 expression in primary microglia, indicating that ICH promotes the transcription and expression of FABP4 in a BACH-1-dependent manner (Fig. 2J-K).

Taken together, our clinical evidence and animal experimental findings indicate a potential role for FABP4 in secondary brain injury after ICH.

### Microglial Fabp4 deficiency attenuates brain damage and promotes neurological recovery after ICH

**Fig. 3:**
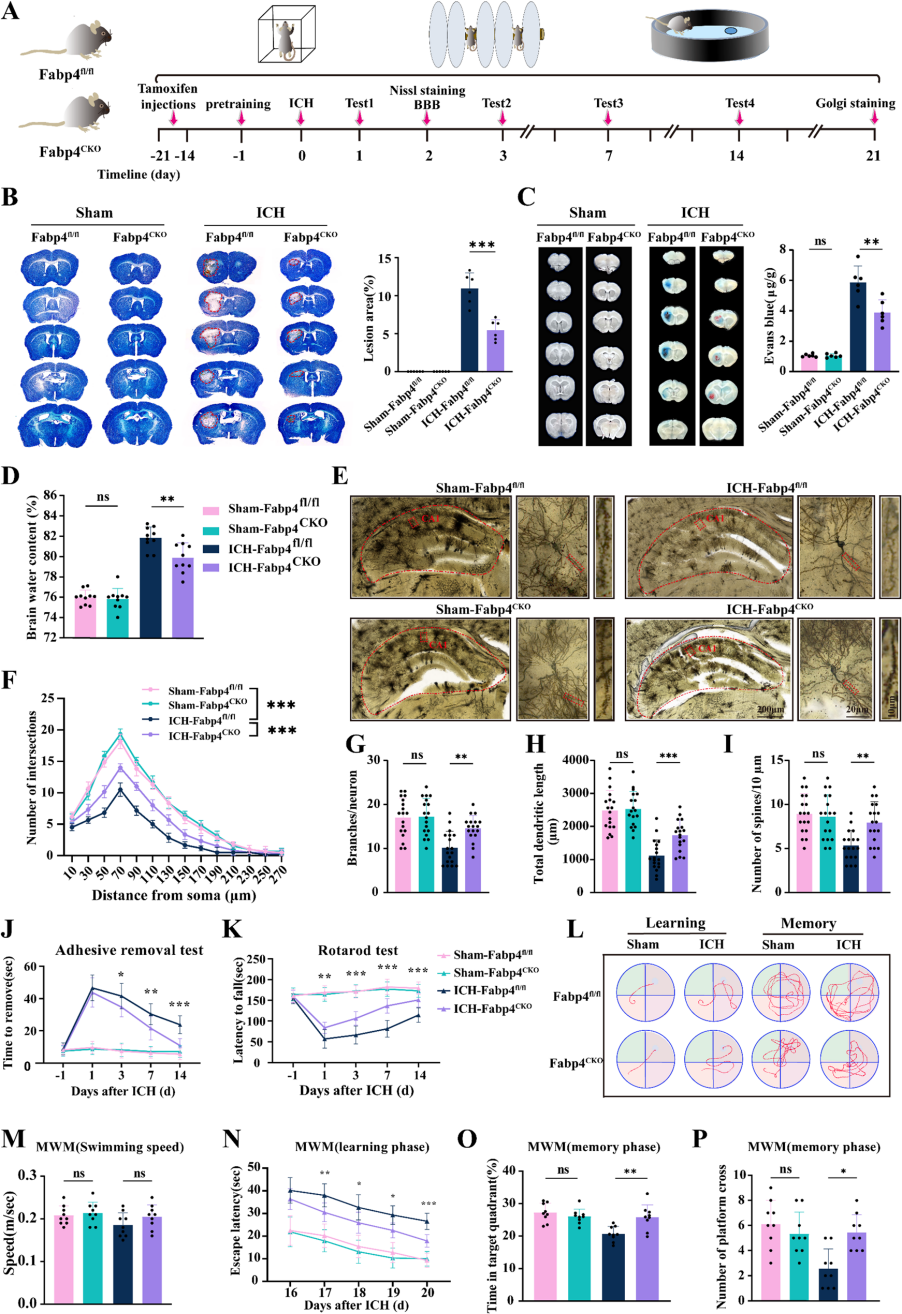
The conditional deletion of Fabp4 in microglia could preserve neuronal health and facilitated neurological recovery.** A** Experiment outline. **B** Representative Nissl staining images gained from each group at 2 d after ICH and the quantification of lesion volumes. Red lines delineate lesion volumes (*n* = 6 per group, two-way ANOVA). **C** Brain sections stained with Evan blue illustrate varying levels of dye leakage in each group two days post-ICH, along with the quantification of Evan Blue for each group (*n* = 6 per group, two-way ANOVA). **D** Measurement of brain water content at 2 days after ICH (*n* = 10 per group, two-way ANOVA). **E-I** Representative Golg staining images demonstrating neurons in the CA1 region of ipsilateral hippocampal at day 21 after ICH, followed by the analysis of intersections (F), branches (G), dendritic length (H) as well as spine numbers (I) using Sholl analysis. (eighteen neurons from 6 animals/group, two-way ANOVA). **J-P** The neurological function of mice was determined through behavioral tests, including the adhesive test, rotarod test, and morris water maze (MWM) test (*n* = 9 per group, J, K, M, N and O for two-way ANOVA, P for Kruskal-Wallis H test). Data are presented as means ± SD. *p < 0.05, **p < 0.01, ***p < 0.001. In behavioral tests, the asterisks above denote single-day comparisons between the two groups (ICH-Fabp4^flox/flox^ group vs. ICH-Fabp4^CKO^ group)

To elucidate the role of FABP4 modulation in the effects of microglia on brain damage after ICH, Nissl staining, Evans blue staining, Golgi staining, brain water content analysis, and behavioral assessments were conducted (Fig. 3A). We generated Fabp4flox/flox: TMEM119creER/+ mice by crossing Fabp4-floxed mice with TMEM119 creERT2 mice. Then, tamoxifen administration was used to induce the conditional microglial knockout of Fabp4 in these mice (Fabp4^CKO^). To confirm the specificity of the knockout, we isolated microglia, brain macrophages, and blood monocytes from ICH-Fabp4^flox/flox^ and ICH-Fabp4^CKO^ mice. We found that FABP4 expression was not altered in brain macrophages or blood monocytes from ICH-Fabp4^CKO^ mice compared to Fabp4^flox/flox^ mice (Figure S2A, Supporting Information), confirming that the deletion of Fabp4 is specific to microglia. Nissl staining revealed significantly smaller ICH-induced brain lesion volumes in the Fabp4^CKO^ mice than in the Fabp4^flox/flox^ controls (Fig. 3B). Evans blue staining indicated that BBB damage was significantly mitigated upon Fabp4 deficiency in microglia (Fig. 3C). In line with these findings, the brain water content analysis indicated that the conditional knockout of Fabp4 mitigated ICH-induced edema (Fig. 3D). Previous studies have reported that continuously progressing microglial inflammation following ICH leads to damage to hippocampal neuronal plasticity and exacerbates neurological dysfunction [25, 26]. Golgi staining revealed that ICH induced a reduction in spine density, dendritic complexity and neurite branching in the CA1 region of the ipsilateral hippocampus at 21 days. However, these pathological alterations were attenuated through microglial Fabp4 genetic ablation (Fig. 3E-I). Additionally, to evaluate the consequences of microglial Fabp4 ablation on neurological outcomes post-ICH, a battery of assessments of sensorimotor integration and cognitive performance, including the adhesive removal test, rotarod test and MWM test, were conducted. In the adhesive removal test, Fabp4^CKO^ ICH mice exhibited shorter adhesive tape removal latency for the affected forelimb on postoperative days 1, 3, 7 and 14 than did Fabp4^flox/flox^ ICH mice (Fig. 3J). Furthermore, ICH mice demonstrated significantly decreased mean fall latency during the rotarod test. Notably, Fabp4^CKO^ ICH mice exhibited prolonged rotarod retention times relative to Fabp4^flox/flox^ ICH mice (Fig. 3K). Moreover, Morris water maze (MWM) assessments were conducted to evaluate whether microglial FABP4 modulation could mitigate ICH-induced cognitive deficits (Fig. 3L). No intergroup differences in swimming velocity were observed (Fig. 3M). During the learning phase, Fabp4^flox/flox^ ICH mice exhibited longer escape latencies than did Fabp4^CKO^ ICH mice (Fig. 3N). In probe trials, Fabp4^CKO^ ICH mice demonstrated increased target quadrant occupancy and platform crossings relative to Fabp4^flox/flox^ ICH mice (Fig. 3O-P). Collectively, these findings indicate that microglial Fabp4 interference attenuates neuronal damage and facilitates neurological recovery after ICH.

### Fabp4 knockout mitigates the microglial inflammatory response and neurotoxicity after ICH

**Fig. 4:**
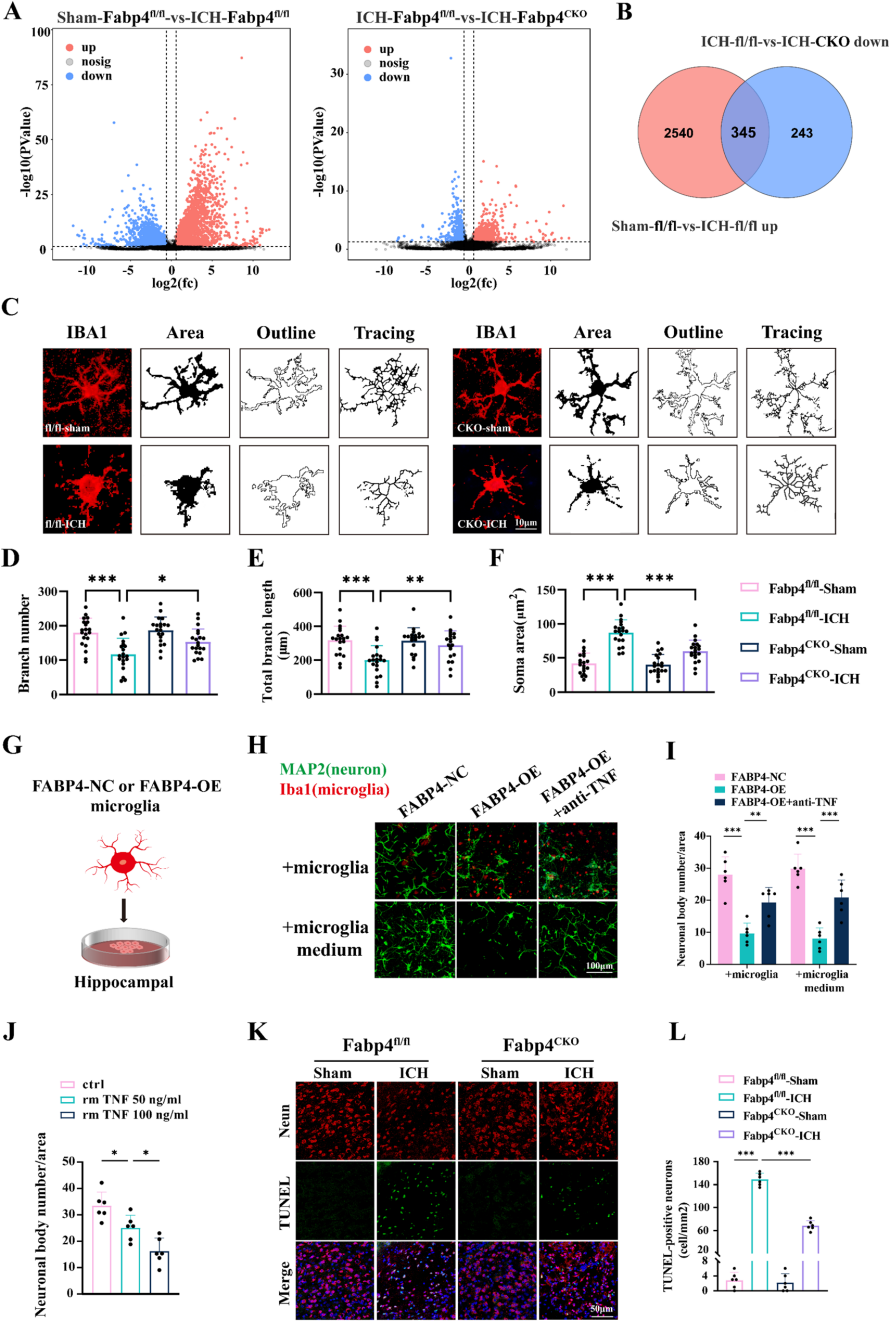
Fabp4 knockout mitigates microglial inflammatory response and neurotoxicity after ICH. **A** The volcano plot clearly displayed the distribution of different genes. **B** The Venn diagram indicated that Fabp4^CKO^ altered the expression of 345 genes. **C** Skeleton analysis of microglia morphologies in Iba1 stained tissue. Scale bar:10 μm. **D**-**F** Statistical analysis of branch numbers, total branch length, and soma area of Iba1-positive cells in each group (*n* = 20 from 5 mice per group, one-way ANOVA). **G**-**H** Representative images of FABP4-OV microglia or their conditioned medium in co-culture with neurons. Scale bars:100 μm. **I** Quantification of the number of neuronal cell bodies (*n* = 6 per group, one-way ANOVA). **J** The relative survival of MAP2 + neurons was observed with increasing doses of recombinant TNF (untreated *n* = 6, 50 ng/ml *n* = 6, 100 ng/ml *n* = 6 FOV, one-way ANOVA). **K**-**L** Images representing TUNEL-positive cells in mouse brain sections were analyzed statistically. Scale bar:50 μm. Data are represented as the quantification of TUNEL positive neurons (*n* = 6 per group, one-way ANOVA). Data are presented as means ± SD. *p < 0.05, **p < 0.01, ***p < 0.001

To clarify the function of FABP4 in the molecular pathophysiology of ICH, RNA sequencing (RNA-seq) at 48 h after ICH was performed to characterize the differential transcriptomic profile associated with FABP4. Volcano plots effectively illustrated the distribution of differentially expressed genes (DEGs) (Fig. 4A). Venn diagram analysis revealed that compared with the sham group, the ICH group presented 2885 DEGs, with 345 of these genes showing reversal following Fabp4 knockout (Fig. 4B). Among the DEGs were disease-associated microglia (DAM)-associated genes such as Fth1, Sparc, Slc2a1, Cd34, Chst2, Ifi44, and Tnfsf8; genes related to endothelial adhesion and neutrophil infiltration, including Pecam1, Esam, and Icam2; as well as inflammation-related genes such as Tnfsf10, S100A10, and Lcn2. Additionally, genes involved in lipid metabolism (e.g., Fabp7, Apod) and mitochondrial function (e.g., PGAM5, Gpx8) were also identified, highlighting the broad and complex biological processes mediated by microglia. Gene Ontology (GO) analysis revealed that among the 345 genes that were regulated by FABP4, immune- and inflammation-related genes accounted for the majority. Heatmap analysis revealed predominant enrichment of DEGs within these pathways. These findings suggest that Fabp4 activity in microglia contributes to ICH-induced neuroinflammation (Figure S3A-B, Supporting Information). Similarly, morphological and fractal analyses revealed that microglia subjected to ICH displayed pronounced retraction, decreased ramification, and increased cell size, indicating a marked shift toward a reactive state. In contrast, these morphological alterations were significantly mitigated in the absence of microglial FABP4 expression (Fig. 4C-F). Given the association between distinct microglial phenotypes and their cytotoxic or neuroprotective effects, the effects of Fabp4 knockout on microglial phenotype modulation were further investigated. Fabp4^CKO^ led to a notable reduction in the number of pro-inflammatory microglia and an increase in the number of anti-inflammatory microglia following ICH (Figure S4A-D, Supporting Information). Collectively, these findings suggest that FABP4 is an important factor that drives the inflammatory polarization of microglia after ICH.

Neurotoxic characteristics are demonstrated by pro-inflammatory microglia [27, 28]. To assess the relative neurotoxic potential, we compared FABP4-overexpressing microglia with control microglia. Primary mouse neurons were cocultured with FABP4-overexpressing (FABP4-OE) and wild-type microglia. Compared with wild-type microglia, FABP4-OE microglia significantly decreased the number of surviving neuronal cells. The exposure of primary neurons to conditioned medium harvested from FABP4-OE microglia decreased the number of surviving neuronal cells. These observations suggest that microglial FABP4 promotes neurotoxicity through soluble factor secretion. Emerging evidence has demonstrated that FABP4 mediates the upregulation of TNF expression [29], which affects neurotoxicity and endothelial activation in multiple neurodegenerative models [30, 31]. The administration of TNF-neutralizing antibodies markedly attenuated FABP4-OE microglia-induced neuronal apoptosis, confirming the pivotal role of TNF in mediating FABP4-driven neurotoxicity (Fig. 4G-J). Consistent with these findings, in vivo TUNEL staining revealed that ICH induced neuronal cell apoptosis in Fabp4^flox/flox^ mice, which was significantly attenuated in Fabp4^CKO^ mice (Fig. 4K-L). Taken together, these findings suggest that FABP4 contributes to the microglial inflammatory response and neurotoxic effects after ICH.

### FABP4 leads to lipid droplet accumulation and enhances protein stability of S100A9

**Fig. 5:**
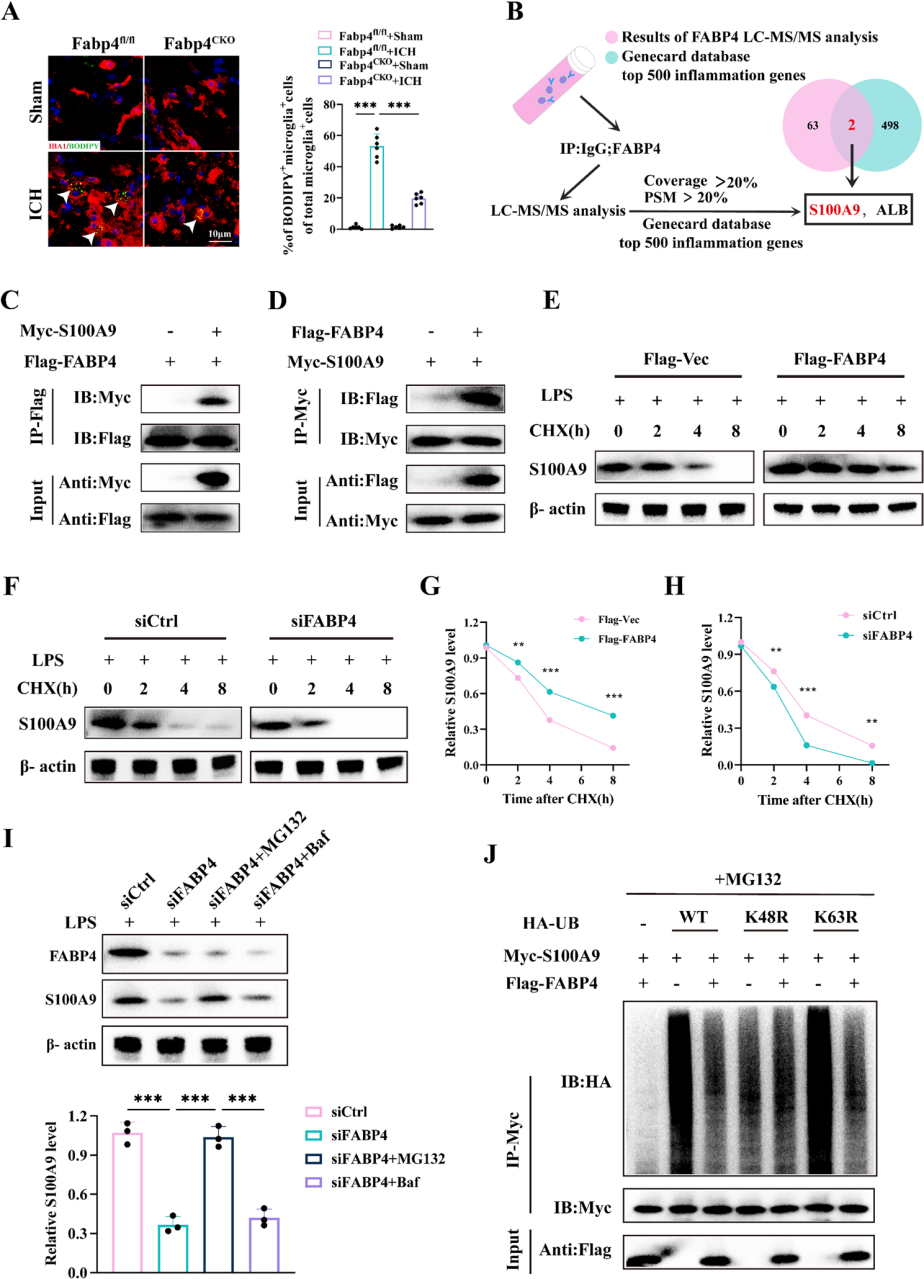
FABP4 drives the buildup of lipid droplets and enhances S100A9 protein stabilization**. A** Representative BODIPY (green), Iba1 (red), and DAPI (blue) co-immunostaining in mice brain Sect. 48 h after ICH. Scale bar: 10 μm. Quantification of BODIPY signal within Iba1 cells (*n* = 6 per group, one-way ANOVA). **B** HEK293T cells received a transfection with a plasmid expressing FABP4 or a control vector. The proteins were enriched via immunoprecipitation and examined using IP–mass spectrometry. These findings were then compared with the top 500 inflammation-related genes from the GeneCards database. **C-D** The experiment involved transfecting HEK293T cells with Myc-S100A9 or a control vector, together with Flag-FABP4. The cellular extracts were immunoprecipitated using an anti-Flag antibody or anti-Myc antibody, subsequently analyzed by Western blot with anti-Myc or anti-Flag antibodies. **E-H** S100A9 protein stability was examined after overexpression of FABP4 and silence of FABP4 in primary microglia treated with CHX and stimulated with LPS. And the quantification of S100A9 protein (*n* = 3 per group, two-way ANOVA). **I** Analysis of FABP4 and S100A9 in FABP4-silenced primary microglia treated with the proteasome inhibitor MG132 or the lysosome inhibitor Baf following LPS challenge. (*n* = 3 per group, one-way ANOVA). **J** Quantification of HEK 293 T cells co-transfected with Flag-FABP4, Myc-S100A9, and the indicated HA-Ub, K48R-only, or K63R-only plasmids, and assessment of S100A9 ubiquitination (*n* = 3). Data are presented as means ± SD. *p < 0.05, **p < 0.01, ***p < 0.001

Next, we explored how FABP4 may lead to the inflammatory state of microglia. Prior studies have indicated that the accumulation of lipid droplets within microglia is closely linked to heightened inflammatory activity, characterized by abnormal lipid storage, impaired phagocytosis, dysfunctional mitochondria, and elevated reactive oxygen species (ROS) levels [32, 33]. ICH markedly increased the accumulation of lipid droplets in microglia. Given the crucial role of FABP4 in fatty acid trafficking, we speculated that the increase in FABP4 expression may be one of the causative factors contributing to the accumulation of lipid droplets. As expected, FABP4 knockout reduced the ICH-induced accumulation of lipid droplets inside microglia (Fig. 5A). Moreover, we employed the acyl-CoA synthetase long-chain family member 1 (ACSL1) inhibitor Triacsin C (TrC), a pharmacological intervention proven to suppress lipid accumulation [34, 35], and demonstrated that the pharmacological inhibition of lipid droplet biogenesis in primary microglia attenuated FABP4 overexpression-induced proinflammatory activation (Figure S5A-C, Supporting Information). These results suggest that the abnormal accumulation of lipid droplets caused by FABP4 may be one of the reasons for the transition to a pro-inflammatory microglial phenotype.

Moreover, immunoprecipitation coupled with mass spectrometry (IP–MS) was used to identify proteins that interact with FABP4. The results were subsequently intersected with the top 500 inflammation-related genes from the GeneCards database, revealing S100A9 as a key interacting protein (Fig. 5B). S100A9, a canonical inflammatory mediator, is actively released during inflammatory processes and orchestrates immune responses through stimulating leukocyte recruitment and inducing cytokine secretion [36, 37]. Supplementary coimmunoprecipitation experiments performed in HEK293T cells with epitope-tagged constructs validated these interactions. Strong coprecipitation of Flag-tagged FABP4 with Myc-tagged S100A9 was detected (Fig. 5C-D). When the protein synthesis inhibitor cycloheximide was added to LPS-treated primary microglial cultures, FABP4 overexpression strongly hindered S100A9 degradation (Fig. 5E-H). These findings revealed that FABP4 is directly associated with S100A9 and influences its stability and degradation, which highlights its role in a regulatory process in microglia. Protein degradation primarily occurs via two pathways: ubiquitination and autophagy. To elucidate the degradation pathways regulating the interaction between FABP4 and S100A9, we performed systematic protein stability assays in FABP4-silenced primary microglia with the proteasome inhibitor MG132 and the lysosome inhibitor Baf following LPS challenge and found that FABP4 did not increase S100A9 protein levels in cells treated with MG132 but did increase S100A9 protein levels in cells treated with the lysosome inhibitor (Fig. 5I). These findings indicate that FABP4 inhibits the ubiquitination–proteasome degradation of S100A9. To validate the role of FABP4 in S100A9 ubiquitination, HEK293T cells were cotransfected with MycS100A9, Flag-tagged FABP4, and HA-tagged ubiquitin. This experiment focused on evaluating how FABP4 affects the polyubiquitination of S100A9. The findings revealed that FABP4 specifically cleaved the K48-linked polyubiquitin chain (Fig. 5J). These data collectively indicate that FABP4 interferes with the ubiquitination and degradation of S100A9 in microglia.

Thus, FABP4 upregulation not only promotes lipid droplet deposition in microglia but also inhibits S100A9 ubiquitination-mediated degradation, synergistically amplifying neuroinflammatory responses.

### Microglial Fabp4 knockout alleviates neutrophil transendothelial migration (TEM) after ICH

**Fig. 6:**
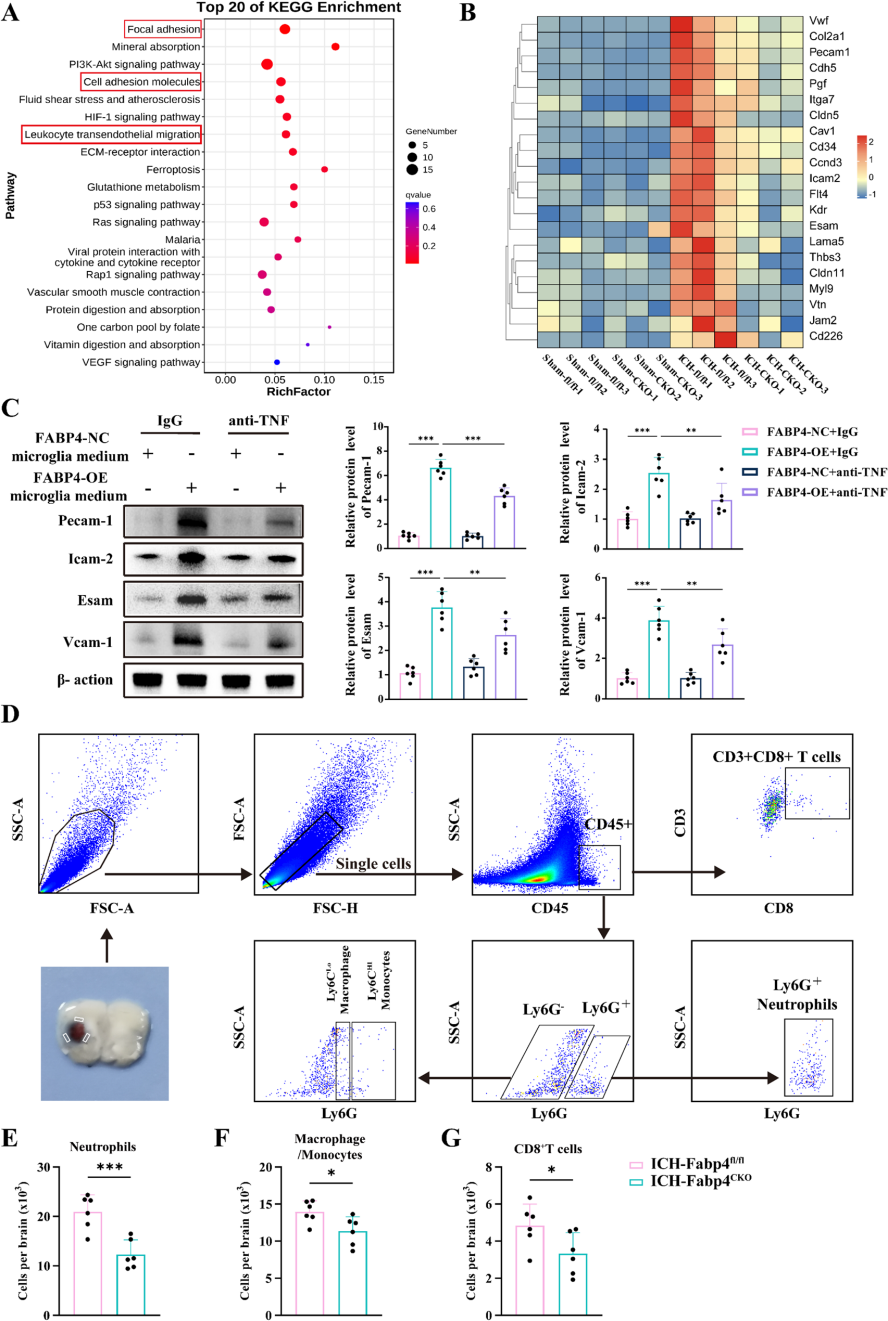
Genetic ablation of microglial Fabp4 attenuates endothelial cell CAMs expression and neutrophil TEM following ICH.** A** KEGG pathway enrichment analysis was performed on the 345 overlapping genes that were identified in the Venn diagram in Fig. 4B. **B** The heatmap displays the expression changes of relevant genes in three distinct pathways enriched in KEGG. **C** Western blot assay of the expression of CAMs including Pecam-1, Icam2, Esam, and Vcam-1 at 48 h post-ICH with IgG and anti-TNF treatments and the related statistical analysis (*n* = 6 per group, one-way ANOVA). **D** Example of a gating strategy used in flow cytometry to analyze immune cells in the brain two days after ICH. Singlets were gated on FSC-A versus FSC-H. CD45 + immune cells were gated based on CD45 staining versus SSC. **E-G** Using cell surface antibodies to distinguish various cell types including Ly6G + neutrophils (E), Ly6G⁻Ly6C⁺ macrophages/monocytes (F), CD8 + T lymphocytes (G) (*n* = 6 per group, Student’s t-test). Data are presented as means ± SD. *p < 0.05, **p < 0.01, ***p < 0.001

Under physiological conditions, brain microvascular endothelial cells (BMECs) serve as gatekeepers, preventing the entry of peripheral immune cells into the brain. However, shortly after the onset of acute brain injury, such as ICH, BMECs typically become activated and exhibit increased expression of cell adhesion molecules (CAMs). These CAMs are crucial for assisting circulating immune cells in adhering to activated BMECs, enabling their TEM into brain tissue [38]. Notably, our RNA-seq analyses revealed significant upregulation of genes associated with the CAM and leukocyte TEM pathways following ICH, whereas microglia-specific FABP4 knockout markedly attenuated these alterations (Fig. 6A-B). These findings suggest that FABP4 activity in microglia modulates endothelial activation and the expression of CAMs. To investigate how microglial FABP4 activity affects endothelial cell CAM expression, we first cultured mouse endothelial cells with conditioned medium from FABP4-OE microglia. This treatment significantly increased CAM expression (Fig. 6C). These findings were consistent with the in vivo results, indicating that microglial FABP4 activity can promote endothelial CAM expression. Previous studies have shown that TNF is a classical stimulator that induces endothelial cell activation and CAM expression [39–41]. Concurrently, our experiments revealed that TNF is among the principal neurotoxic substances released through microglial FABP4 activity. As expected, TNF-neutralizing antibodies effectively reversed the elevated CAM levels induced by FABP4-OE microglia.

We subsequently investigated whether the conditional knockout of FABP4 affects the infiltration of peripheral immune cells into the brain, as emerging evidence highlights the critical role of peripheral immune responses in pathological processes following ICH. Initial gating revealed CD45high and CD45int populations (Fig. 6D). Subsequent analyses revealed neutrophils (CD45high Ly6G+), macrophages/monocytes (CD45high Ly6G-Ly6C), and T cells (CD45high CD3e + CD8+) (Fig. 6E-G). Compared with those in the Fabp4^flox/flox^ ICH group, the neutrophil frequency in the Fabp4^CKO^ ICH group was significantly lower. We also isolated Ly6G⁺ neutrophils from sham and ICH mice and found that FABP4 expression remained unaltered in neutrophils from ICH mice compared to sham controls (**Figure** S6A, Supporting Information). This confirms that the observed reduction in neutrophil infiltration in ICH-Fabp4CKO mice is directly attributable to microglial FABP4 deletion. These results suggest that microglial Fabp4 knockout alleviates neutrophil TEM after ICH.

### Neutrophil-mediated overproduction of neutrophil extracellular traps (NETs) aggravates ICH-induced brain injury

**Fig. 7:**
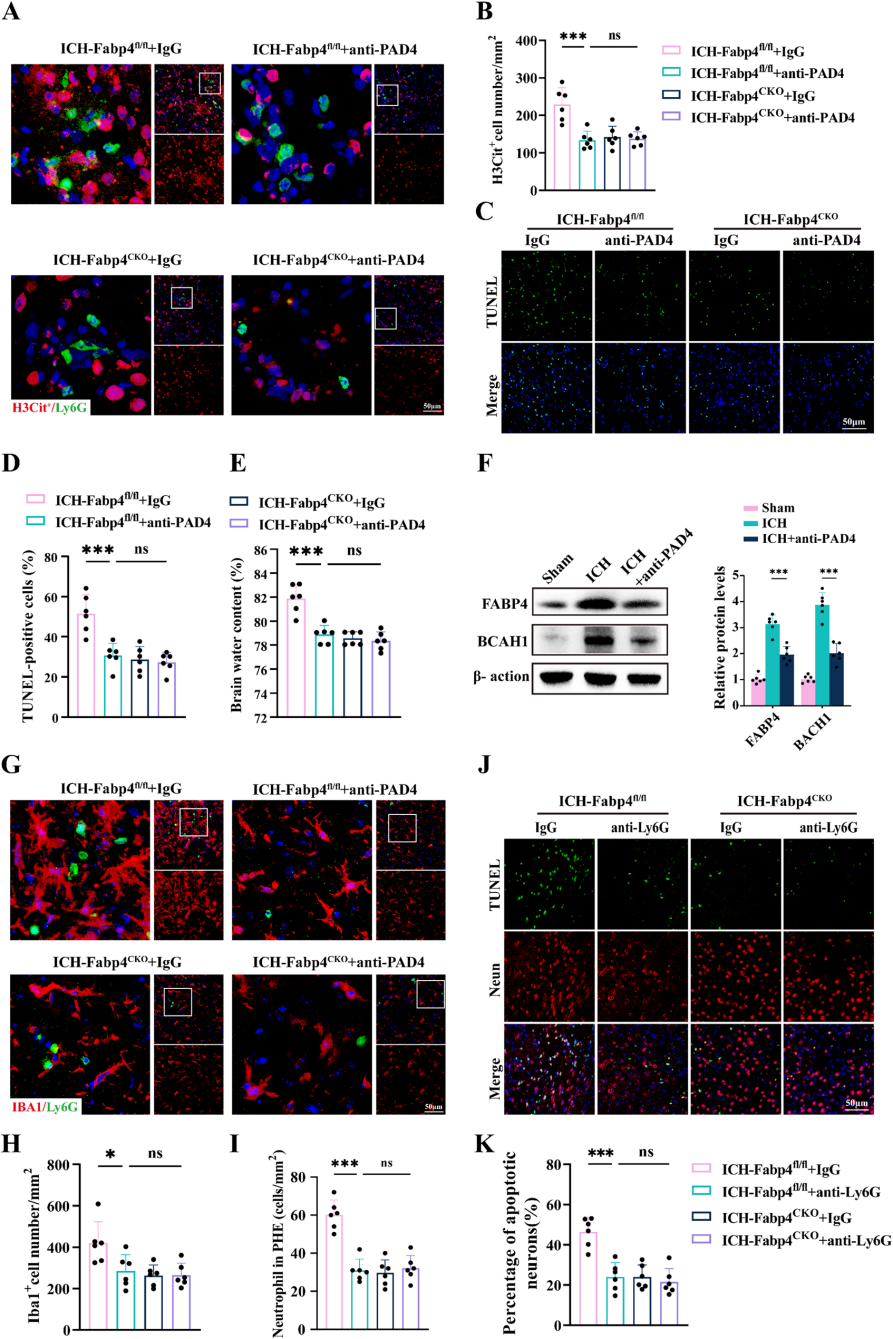
Excessive formation of NETs exacerbates brain injury caused by ICH. **A** Immunofluorescence staining of H3-cit (red) and Ly6G (green) was performed on Fabp4^flox/flox^ and Fabp4^CKO^ mice following various treatments. Scale bar:50 μm. **(B)** Quantification analysis of the H3-cit cell number per mm^2^ (*n* = 6 per group, one-way ANOVA). **C-D **Brain sections of mice showed representative images and statistical analysis of TUNEL-positive cells 48 h after ICH in each group. Scale bar:50 μm. *n* = 6 per group, one-way ANOVA. **E** Quantification of brain water content at 48 h after ICH (*n* = 6 per group, one-way ANOVA). **F** Western blot assay of the effects of anti-PAD4 on the expression of FABP4 and BACH1 at 48 h after ICH in mice and the related statistical analysis (*n* = 6 per group, one-way ANOVA). **G-I** Immunofluorescence staining of Iba1 (red) and Ly6G (green) was conducted on Fabp4^flox/flox^ and abp4^CKO^ mice. Scale bar:50 μm. H) Quantification analysis of the Iba1 cell number per mm^2^ and neutrophil in PHE per mm^2^ (*n* = 6 per group, one-way ANOVA). **J-K** Images and statistical data of TUNEL-positive cells in mouse brain sections are shown (*n* = 6 per group, one-way ANOVA). Data are represented as the percentage of TUNEL-positive cells. Scale bar: 50 μm. Data are presented as means ± SD. *p < 0.05, **p < 0.01, ***p < 0.001

Neutrophils have been reported to exacerbate neural damage primarily through the secretion of NETs. We found that ICH led to elevated numbers of H3Cit + neutrophils and increased NET formation, which was mitigated by FABP4 deficiency. To further investigate the roles of neutrophils and NETs in ICH-related brain injury, we treated cells with the PAD inhibitor Cl-amidine to inhibit NET formation (Fig. 7A-B). This approach significantly reduced the proportion of TUNEL-positive cells and brain edema in FABP4^flox/flox^ ICH mice but did not confer additional protective effects in FABP4^CKO^ ICH mice (Fig. 7C-E). Collectively, these findings suggest that microglial Fabp4 knockout alleviated endothelial inflammatory activation and neutrophil TEM following ICH.

Interestingly, we also found that NETs further promoted the expression of FABP4 in microglia, increased microglial activation, and increased neuronal loss (Fig. 7F-K). These findings highlight a positive feedback pathway between microglial FABP4 activity and neutrophils. In summary, microglial Fabp4 knockout appears to alleviate brain damage, at least partially, by reducing neutrophil infiltration and NET generation.

### VCAM-1-targeting mesoporous silica nanoparticles (MSNs) effectively deliver siFABP4 to pathologically affected brain tissue

**Fig. 8:**
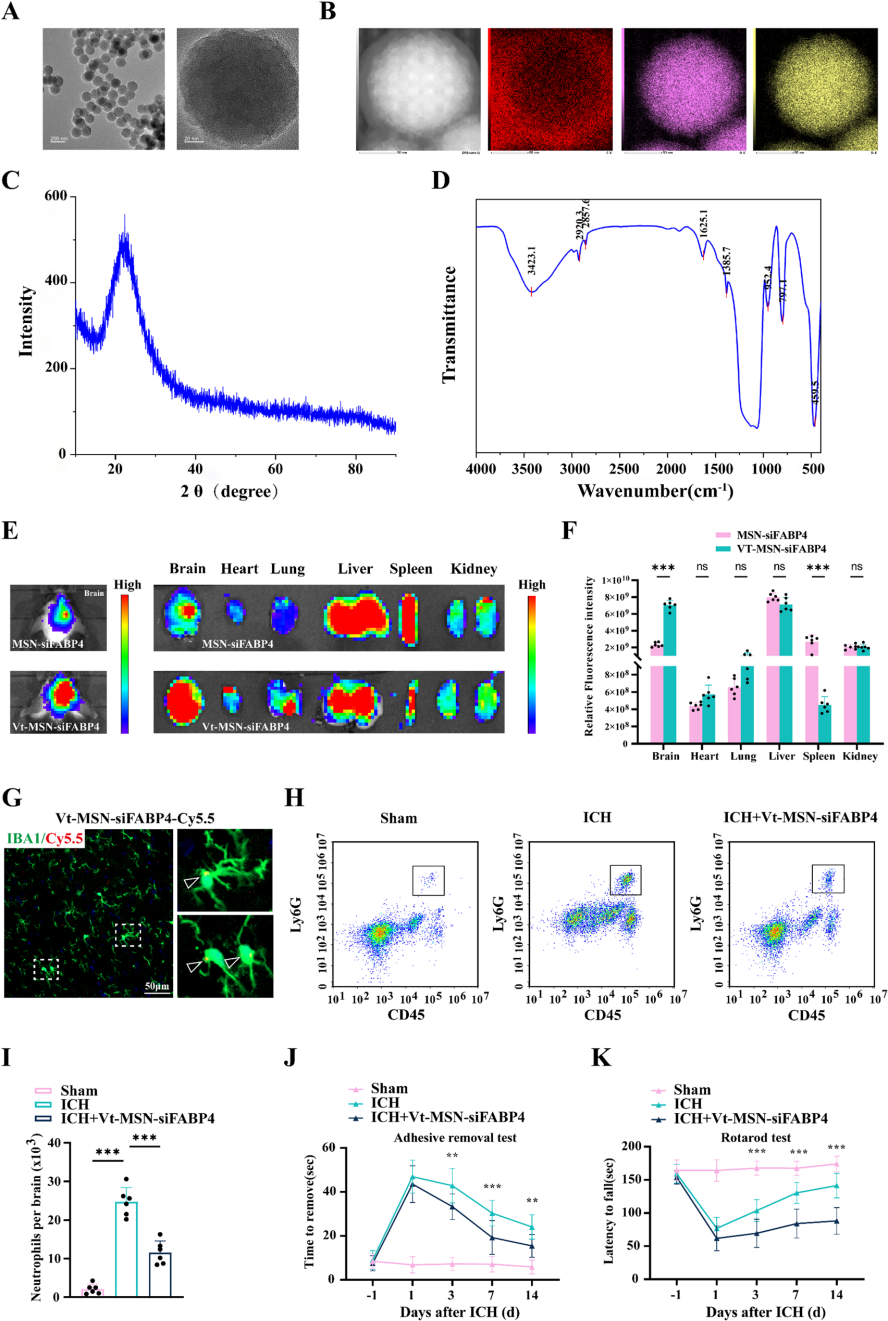
VCAM-1 targeted MSN delivery of siFABP4 attenuates neuroinflammation and promotes neurological recovery after ICH. **A**-**D** Transmission electron microscopy (TEM) (**A**), X-ray Diffraction (XRD) (**B**), Energy-dispersive X-ray spectroscopy (EDS) (**C**), Fourier-Transform Infrared Spectroscopy (FTIR) (**D**) of Vt-MSN-siFABP4. **E-F** Real-time fluorescence imaging and analysis reveal the distribution of Cy5.5-labeled non-targeting MSN-siFABP4 or Vt-MSN-siFABP4 in ICH mice 24 hours after intravenous injection (*n* = 6 per group, Student’ s t-test). **G** The images depict Iba1-stained (green) brain sections from mice treated with Cy5.5 (red) 6 h after injection. Scale bar:50 μm. **H-I** Flow cytometry was used to analyze how Vt-MSN-siFABP4 affects the infiltration of Ly6G + neutrophils in the brain two days after ICH in mice (*n* = 6 per group, one-way ANOVA). **J-K** The sensorimotor abilities of the animals were evaluated using the adhesive test (J) and the rotarod test (K) (*n* = 12 per group, two-way ANOVA). Data are presented as means ± SD. *p < 0.05, **p < 0.01, ***p < 0.001

These experimental findings strongly indicate that FABP4 serves as a critical mediator of the pathogenesis of secondary brain injury following ICH. Building upon this discovery, we developed a nucleotide-based therapeutic strategy targeting FABP4 for ICH intervention. To address the inherent instability of siRNA molecules, we engineered an MSN-based delivery system that effectively encapsulates FABP4-specific siRNA. To enhance lesion-specific delivery, we implemented a targeting modification strategy based on pathophysiological changes in the brain. Building on our previous observation of significant upregulation of CAMs, particularly VCAM-1, on cerebral vascular endothelium after ICH, we functionalized the MSN surface with a high-affinity targeting peptide (VHPKQHR) through carbodiimide crosslinking chemistry. Comprehensive characterization of the resulting VCAM1-targeted, FABP4 siRNA-loaded MSNs (Vt-MSN-siFABP4) was performed using multiple analytical techniques, including transmission electron microscopy (TEM), X-ray diffraction (XRD), energy-dispersive X-ray spectroscopy (EDS) and Fourier transform infrared spectroscopy (FTIR) (Fig. 8A-D). We then examined whether Vt-MSN-siFABP4 might be capable of overcoming the blood–brain barrier (BBB) barrier. Mice were intravenously injected with Cy5.5-labeled MSN-siFABP4 or Vt-MSN-siFABP4, followed by live fluorescence imaging analysis. At 24 h after intravenous injection, the concentration of Cy5.5-labeled Vt-MSN-siFABP4 in the brain was greater than that of MSN-siFABP4 (Fig. 8E-F). These findings suggest that compared with MSN-siFABP4, Vt-MSN-siFABP4 is better at targeting the brain. Moreover, immunofluorescence staining with Cy5.5-labeled Vt-MSN-siFABP4 and Iba1 demonstrated that Vt-MSN-siFABP4 is efficiently taken up by microglia (Fig. 8G).

**Scheme 1:**
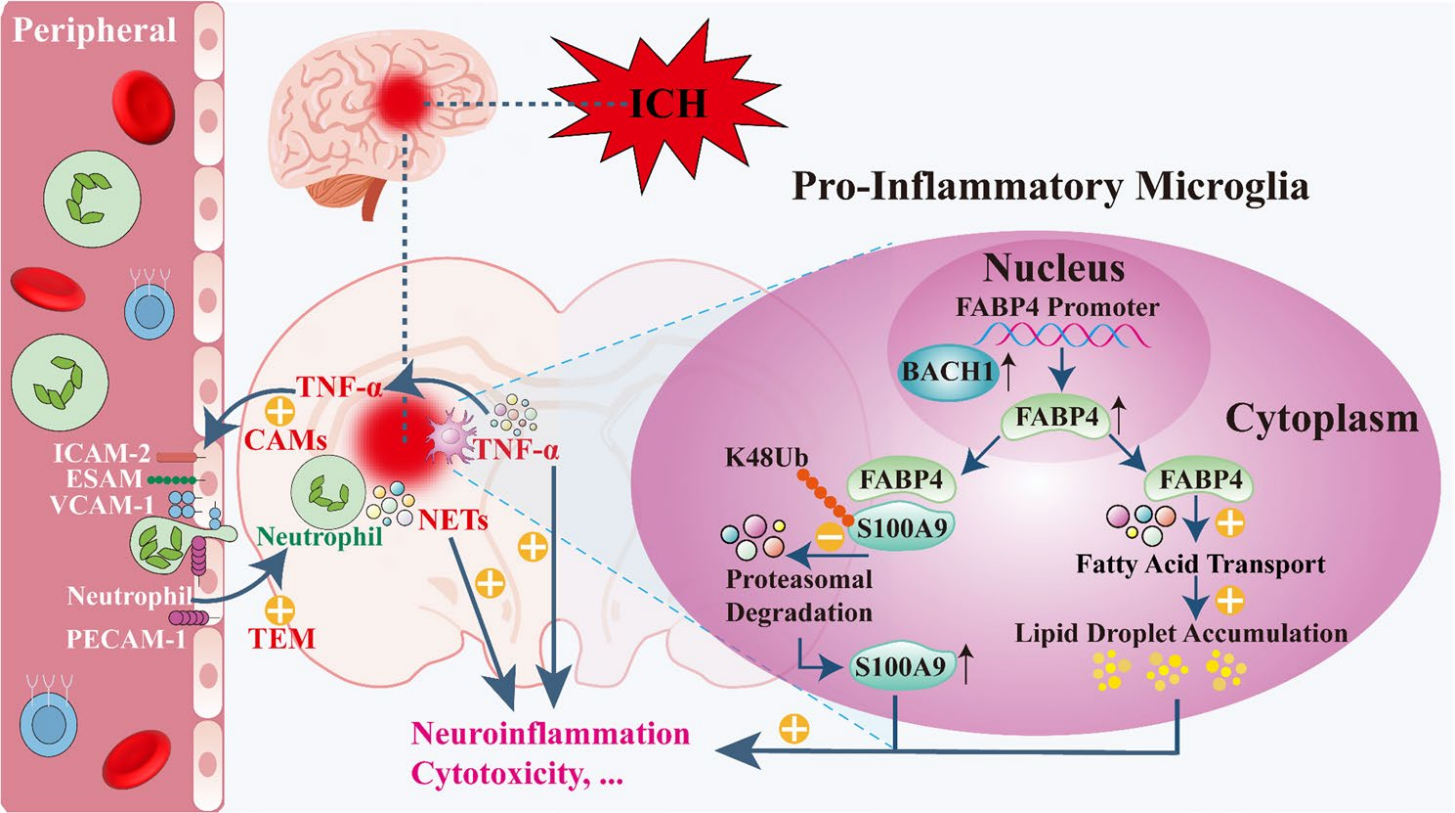
Schematic diagram of FABP4 drives neuroinflammation after ICH

Next, we explored whether Vt-MSN-siFABP4 could reduce brain injury caused by ICH and enhance functional recovery in mice. Two days after ICH, microglial activation and neutrophil infiltration were significantly reduced following Vt-MSN-siFABP4 treatment (Figure S7A, Supporting Information; Fig. 8H-I). This therapeutic intervention markedly decreased the TUNEL-positive cell ratio and brain edema in ICH mice (Figure S7B-D, Supporting Information). Similarly, behavioral assessments indicated that Vt-MSN-siFABP4 therapy enhanced sensorimotor ability in ICH mice (Fig. 8J and K). Collectively, these results suggest that Vt-MSN-siFABP4 treatment aids in recovery after ICH.

## Discussion

Through integrated analysis of clinical samples and systematic validation via in vivo and in vitro experimental models, this study provides the first mechanistic evidence implicating FABP4 as a central orchestrator of neuroinflammatory cascades and secondary injury progression following ICH. We demonstrate that ICH triggers the BACH1-mediated transcriptional upregulation of FABP4 in activated microglia. Mechanistically, we found that FABP4 initiates an inflammatory response through two synergistic pathways: (i) promoting lipid droplet biogenesis and (ii) stabilizing S100A9 by inhibiting its K48-linked ubiquitination. Crucially, FABP4-overexpressing microglia not only exhibit intrinsic neurotoxicity but also promote neutrophil TEM through endothelial activation, exacerbating cerebral damage. The proposed mechanistic pathway is schematically depicted in Scheme 1. The clinical relevance of these findings is underscored by the significant correlations between plasma FABP4 levels and neurological outcomes in ICH patients. To bridge translational gaps, we developed brain-targeted nanoparticles encapsulating FABP4-specific siRNA, which demonstrated enhanced BBB penetration and therapeutic efficacy in preclinical models.

While microglial dysregulation is recognized as a key contributor to secondary brain injury, the transcriptional drivers of their pathogenic transformation remain poorly defined. Our discovery of BACH1-mediated FABP4 induction addresses this knowledge gap. The observed neuroprotective effect in microglial Fabp4-knockout models—evidenced by attenuated neuronal loss, decreased edema, and functional recovery—suggests that FABP4 is a critical mediator of post-ICH damage.

Recent research has shown that lipid droplets play a dual role in influencing microglial inflammatory responses, especially in acute ischemic stroke and neurodegenerative diseases [42, 43]. In acute ischemic stroke, microglia accumulate LDs during the early phase, which is associated with increased phagocytic activity and transcriptional signatures linked to inflammation. Intriguingly, pharmacological inhibition of LD lipolysis via Atglistatin, an ATGL inhibitor, reduced proinflammatory cytokine (e.g., TNF-α and IL-6) production and improved neurobehavioral outcomes in mouse models of middle cerebral artery occlusion (MCAO), suggesting that LDs may act as reservoirs to sequester proinflammatory lipids and mitigate excessive neuroinflammation. However, the role of LDs appears context dependent. For instance, under aging or chronic neuroinflammatory conditions, LD-laden microglia exhibit proinflammatory phenotypes and impaired phagocytosis, exacerbating neurodegeneration. These results emphasize the therapeutic possibilities of targeting LD metabolism to balance microglial inflammatory responses, although further research is needed to clarify its divergent roles across disease stages and models. Given the crucial role of FABP4 in fatty acid trafficking, we speculated that an increase in FABP4 expression may lead to the aggregation of lipid droplets and inflammatory reactions in microglia. As expected, Fabp4 knockout reduced the accumulation of lipid droplets inside microglia, suggesting that the abnormal accumulation of lipid droplets caused by increased FABP4 expression may be one of the reasons for the acquisition of a reactive phenotype that promotes inflammation.

The proinflammatory protein S100A9, a key component of the alarmin family, has emerged as a critical mediator of microglial activation in neuroinflammatory and neurodegenerative diseases [44, 45]. In BV-2 microglia, high-dose S100A9 stimulation activates the Toll-like receptor (TLR)−4 and TLR-7 pathways, triggering the release of TNF-α and IL-6, which impair cell viability and amplify neurotoxic inflammation [46, 47]. This finding aligns with its role in Alzheimer’s disease (AD), where S100A9 is implicated in the amyloid–neuroinflammatory cascade, driving chronic microglial activation and neuronal damage. In systemic inflammation, S100A8/S100A9 (calprotectin) also promotes leukocyte infiltration and cytokine production via the NF-κB and MAPK pathways, which are mechanisms conserved in microglial responses [48, 49]. In our study, we identified S100A9 as a novel protein that interacts with FABP4. FABP4 hindered the ubiquitination and degradation of S100A9 in microglia. This may be another pathway through which FABP4 activates inflammation in microglia.

Previous study in ischemic stroke has demonstrated that FABP4 activation can upregulate MMP-9 to disrupt tight junctions and exacerbate BBB injury [22]. Our study in ICH revealed a more comprehensive mechanistic landscape. We found that microglial FABP4 promoted lipid droplet accumulation and inhibited ubiquitin-proteasome-mediated degradation of S100A9 in microglia, synergistically amplifying neuroinflammation. These findings do not preclude a role for MMPs in ICH but rather suggest that FABP4 operates through additional, complementary mechanisms in the hemorrhagic setting, constituting a more complete complement to these established mechanisms.

As the resident immune cells of the CNS, microglia engage in extensive crosstalk with peripheral immune cells and play a critical role in regulating the infiltration of peripheral immune cells into the brain [50]. This infiltration is strongly associated with secondary brain injury, primarily driving neuronal death through the release of proinflammatory cytokines, reactive oxygen species (ROS), matrix metalloproteinases (MMPs), and proteases [51]. A key consideration is that peripheral immune cells must traverse the BBB to enter the brain. In physiological settings, the BBB acts as a selective barrier, efficiently preventing toxins, pathogens, and circulating immune cells from entering the CNS. However, following acute brain injury, BMECs undergo rapid activation, marked by the upregulation of inflammatory CAMs. These CAMs play a crucial role in enabling circulating immune cells to adhere to inflamed BMECs, which aids their TEM through the endothelial barrier into the brain parenchyma [38]. Our RNA-seq data revealed a pronounced upregulation of genes associated with the CAM pathway and leukocyte TEM following ICH, which was markedly attenuated in microglia-specific Fabp4 knockout mice. To further elucidate the role of FABP4 in this process, we examined the effect of Fabp4 conditional knockout on peripheral immune cell infiltration. Notably, we observed a substantial reduction in the frequency of neutrophils in the brains of Fabp4^CKO^ ICH mice compared with those of Fabp4^flox/flox^ ICH control mice. This reduction in neutrophil infiltration is of particular significance, as excessive numbers of brain-infiltrating neutrophils exacerbate microglial activation and brain injury through the overproduction of NETs. NETs, which are composed of DNA‒histone complexes and granular proteins, amplify neuroinflammation by activating microglia and damaging surrounding neural tissue, thereby perpetuating a vicious cycle of secondary injury.

To improve the translational and practical significance of our research, we investigated pharmacological interventions. Although gene-editing techniques targeting FABP4 are effective for experimental purposes, their clinical utility is limited. Additionally, the BBB poses a significant challenge for drug delivery to the brain, necessitating an efficient delivery system. Nanoparticles have shown promise in enhancing BBB penetration and brain targeting [52]. Owing to the susceptibility of nucleotides to degradation, we engineered an MSN-based delivery system that effectively encapsulates FABP4-specific siRNA, protects the contents from degradation and demonstrates superior biocompatibility relative to man-made materials. Moreover, we investigated ways to improve the ability of MSNs to target diseased brain tissue. Significantly, CAMs are abundant in damaged brain tissue following ICH, with VCAM-1 being a notable marker of pathological endothelial cells [53]. Therefore, we altered the MSN surface by binding a targeting peptide (VHPKQHR) known to bind to VCAM-1 to selectively deliver FABP4-specific siRNA to pathological brain tissue [54, 55], which successfully restored brain tissue barrier function, inhibited neutrophil migration, and prevented secondary brain damage in ICH model mice. The Vt-MSN-siFABP4 system facilitated BBB penetration, improved siFABP4 delivery to the brain, and enhanced microglial uptake. This system also mitigated microglial overactivation and exerted anti-inflammatory effects in ICH models. Furthermore, Vt-MSN-siFABP4 significantly alleviated neurological damage and promoted recovery postinjury. Collectively, our findings indicate that the Vt-MSN-siFABP4 system is a promising and clinically viable strategy for neuroprotection in ICH.

However, several limitations of our study should be acknowledged. Firstly, total white blood cell (WBC) count is a composite measure encompassing multiple cell types—including monocytes, neutrophils, and lymphocytes—each potentially playing divergent roles in ICH. Changes in specific subpopulations may thus be masked in the overall WBC count. Although we observed no significant difference in total WBC in our clinical cohort, subtype-specific alterations may still influence disease progression or recovery, a possibility that warrants further investigation. Secondly, it is worth noting that FABP4 was also elevated in infiltrating myeloid population, albeit to a lesser extent than in resident microglia. The specific function of FABP4 in this cell population and its potential interplay with microglial FABP4 warrants further investigation in future studies. Thirdly, while we employed the classic pro-inflammatory/anti-inflammatory framework to demonstrate FABP4’s role in modulating microglial polarization, this binary classification represents a considerable oversimplification of microglial heterogeneity. In future studies, we will utilize single-cell RNA sequencing technologies to gain a more nuanced understanding of how FABP4 expression shapes specific microglial subpopulations and their transcriptomic profiles under neuroinflammatory conditions. Fourthly, while our study establishes that FABP4 promotes TNF production through lipid droplets, the precise interrelationships among lipid droplet accumulation, TNF, FABP4, and S100A9, as well as the exact intracellular signaling pathways involved, remain to be elucidated in future studies. Lastly, while our nanoparticle-based therapeutic strategy demonstrates that targeting microglial FABP4 confers significant therapeutic efficacy in ICH pathogenesis, we cannot fully exclude the involvement of other myeloid populations, such as infiltrating monocytes/macrophages, which may also uptake the nanoparticle and contribute to the overall therapeutic outcome. Future studies using single-cell technologies and conditional knockout models targeting other myeloid lineages will help dissect their distinct functions.

## Materials and methods

### Human blood samples and brain tissues

Human research in the present study was conducted in accordance with Ethics Committee of Tangdu Hospital, the Air Force Military Medical University (K201906-12). Participants or their legal guardians provided written informed consent. In the validation cohort, Eighty ICH patients receiving conservative treatment underwent analysis. The study population comprised adults aged 18–75 years, excluding those with malignancies, acute infections, inflammatory conditions, diabetes, or mental disorders. Six-month neurological outcomes were assessed with mRS, categorizing functional recovery as favorable outcomes (mRS 0–2) and unfavorable outcomes (mRS 3–6). Peripheral blood samples were obtained via antecubital venipuncture within 24 h of symptom onset and immediately anticoagulated with ethylenediaminetetraacetic acid (EDTA). Blood specimens underwent initial centrifugation (2,000 × g, 10 min) and subsequent high-speed centrifugation (10,000 × g, 2 min) for platelet depletion. Processed plasma was cryopreserved at − 80 °C pending biochemical assays [56]. For brain tissue sample collection, adjacent brain tissue surrounding intracerebral hematomas was excised according to established surgical guidelines to alleviate localized pressure and mitigate secondary edema development. This resected material was retained for later examination. Control specimens consisted of histologically verified normal parenchyma obtained during deep tumor resection procedures, confirmed as tumor-free intraoperatively by neurosurgeons. Individuals presenting with autoimmune conditions, neurological disorders, CNS infections, or ongoing immunomodulatory treatments were excluded. Subsequently, PHE was quantified using 3D Slicer software at 72 h after symptom onset, followed by comprehensive analysis.

### Animals and ethics

Adult male wild-type C57BL/6J mice (8 weeks old, 20–25 g) were obtained from the Animal Center of Air Force Medical University, while FABP4-floxp mice were generously provided by Shanghai Model Organisms Center, Inc. To generate FABP4flox/flox: TMEM119creER/+ mice, we crossed FABP4-floxed mice with TMEM119creERT2 mice (Shanghai Model Organisms Center). Conditional microglial knockout of Fabp4 was induced in these mice (designated as Fabp4^CKO^) through consecutive 7-day tamoxifen administration (#S1238, Selleck; 150 mg/kg per day). ICH surgeries were performed 14 days after the final injection. All animals were kept in a pathogen-free environment with unlimited access to food and water. Experimental procedures strictly followed the National Institutes of Health Guidelines for the Care and Use of Laboratory Animals and were approved by the Animal Experiment Center of Air Force Military Medical University (IACUC-20210555). We implemented rigorous measures to minimize both animal suffering and the number of experimental subjects.

### Models of ICH

Mice underwent stereotactic collagenase injection into the right striatum to induce ICH [57, 58]. The procedure consisted of the following steps: After deep anesthesia with 30% chloral hydrate (intraperitoneal administration), mice were secured in a stereotaxic frame (RWD Life Science Co.,LTD). A midline scalp incision was made under aseptic conditions, followed by creation of a cranial burr hole (1 mm diameter) at coordinates 1.0 mm anterior to bregma and 2.1 mm lateral to the right. Then, the syringe was injected to a depth of 3.20 mm within 5 min. After the injection of collagenase, the injection needle remained in situ for 5 min post-injection to prevent reflux, followed by gradual needle withdrawal and skull closure. Sham-operated mice received identical procedures excluding collagenase administration. Core body temperature was maintained at 37 ± 0.5 °C throughout the procedure using a heating pad. Postoperative mortality (15–20%) and mice lacking neurological deficits were excluded from subsequent analyses.

### Quantification of lesion volumes

We quantified lesion volumes in mice 2 days after ICH using Nissl staining. For each animal, five 25-µm-thick frozen sections were collected along the ventrodorsal axis of the hematoma at 125-µm inter-slice intervals. Following Nissl staining, lesion areas were analyzed by an investigator blinded to experimental groups using ImageJ software. The lesion volume was calculated using the following formula:

Lesion Volume (%) = *[Σ(A_n × T)/V_total] × 100*% [59].

where *A_n* denotes hemorrhage area in section n, *T* represents section thickness (0.15 mm) = physical thickness (25 μm) + inter-slice interval (125 μm) and *V_total* is the reference whole-brain volume.

### Golgi-Cox staining

The Golgi-Cox staining procedure was implemented following the manufacturer’s protocol for the FD Rapid Golgi Stain kit (PK401, FD Neuro Technologies). In brief, mouse brains were sequentially immersed in Solutions A + B (14 days) and Solution C (3 days). 100 μm-thick coronal sections were generated using a cryostat, then processed through staining, dehydration, and xylene-mediated clearing. Neuronal reconstruction and analysis were conducted using Fiji-ImageJ with the Simple Neurite Tracer plugin. Each experimental group underwent blinded evaluation of eighteen neurons sampled across six animals.

### Brain water content

Brain water content was quantified using the standard wet-dry method [57]. Following rapid harvest, brain tissues were dissected into three distinct regions: the hemispheric hemorrhage zone, contralateral hemisphere, and cerebellar compartment. All specimens underwent immediate wet weight measurement using analytical-grade balances. Subsequent dehydration was performed in a precision drying oven maintained at 95–100 °C for 72 h to determine dry mass. The tissue hydration percentage was computed using the formula: Water content (WC) was calculated using:

WC (%) *= [(W*_*w*_ *- W*_*d*_*)/W*_*w*_*] × 100*

where *W*_*w*_ represents wet weight and *W*_*d*_ denotes dry weight.

### Neurobehavioral test

As described above, we conducted blinded neurobehavioral assessments in all experimental groups at predetermined time intervals. Three standardized tests were systematically performed as follows:

Adhesive removal test [60]. Sensorimotor responsiveness was quantified by measuring latency to remove adhesive stimuli. A standardized tape strip (2 × 3 mm) was affixed to the left forepaw, with timing initiated at first oral contact and terminated upon complete removal. Trials were capped at 120 s to prevent distress. Preoperative baseline testing preceded postoperative evaluations conducted at days 1, 3, 7, and 14.

Rotarod test [60]. Motor function is assessed by observing and recording the time that mice hold from the beginning of the rotation of the rod until they fall. Following 15-min habituation, mice underwent three consecutive trials on a rod with linear acceleration from 4 to 40 rpm over 300 s. Inter-trial intervals of 5 min prevented fatigue accumulation. Latency to fall (seconds) was automatically recorded via infrared sensors, with mean latency derived from triplicate measurements. Testing chronology paralleled other behavioral assessments, including preoperative baseline determination.

Morris Water Maze Test (MWM) [3]. Spatial cognitive function was evaluated through a standardized acquisition-probe paradigm. The water maze consisted of a circular pool (120 cm diameter) filled with opacified water (22 ± 1 °C, titanium dioxide suspension). Four distinct visual cues were positioned equidistantly on the pool walls. During the acquisition phase (postoperative days 16–20), mice underwent thrice-daily training sessions (30-min inter-trial intervals) to locate a submerged acrylic platform (10 cm diameter).Each trial commenced with the mouse being gently released facing the pool wall at one of four randomized cardinal starting positions Escape latency (time from immersion to platform localization) was recorded with a 60-s cutoff, after which mice were manually guided to the platform for 10-s spatial orientation. The probe trial on day 21 involved platform removal and 60-s free exploration initiated from the quadrant diametrically opposed to the original platform location.

### BBB permeability

To evaluate BBB permeability, Evans blue extravasation was performed 48 h after ICH. Three hours before sacrifice, mice received an intravenous injection of Evans blue (4 mL/kg) via the tail vein. Following deep anesthesia with 2% pentobarbital sodium (lethal dose), animals underwent transcardiac perfusion with 40 mL ice-cold 0.9% saline. After weighing, the brain tissues were divided into six equally spaced coron al slices for macroscopic documentation. The samples were homogenized in saline and then centrifuged at 12,000 ×g for 30 min at 4 °C. The supernatant obtained was combined with an equal amount of trichloroacetic acid and left to incubate overnight at 4 °C. After repeat centrifugation (12,000 ×g, 30 min, 4 °C), Evans blue concentration was quantified spectrophotometrically at 620 nm.

### Tissue preparation

As described in the preceding protocol, at 48 h post-ICH induction, following terminal anesthesia induction, mice underwent transcardiac perfusion with PBS (pH 7.4) and sequential perfusion with 4% paraformaldehyde (PFA). Brains were carefully excised maintaining structural integrity, cryoprotected by immersing them in 30% sucrose solutions. Coronal Sects. (15–25 μm thickness) were prepared using a cryostat (Leica CM1900) and subsequently processed for immunofluorescence microscopy and TUNEL assay.

### Immunofluorescence

In brief, tissue permeabilization was achieved through incubation in PBS supplemented with 3% Triton X-100 and 5% goat serum (Gibco) under ambient conditions for 20 min. Subsequently, Sections were placed in primary antibodies and incubated overnight at 4 °C: anti-IBA1 (1:300; Novus; NB100-1028), anti-GFAP (1:1000; Invitrogen; PA1-10004), anti-NeuN (1:400; Merck; ABN90), anti-FABP4 (1:200; Abcam; ab92501), anti-MAP2 (1:200; Abcam; ab5392), anti-CD86 (1:300; Proteintech; 13395-1-AP), anti-CD206 (1:500; Abcam; ab300621), BODIPY (1:1000; Invitrogen; D3922), anti-Histone H3 (1:500; Abcam; ab300641), and anti-Ly6G (1:200; Proteintech; 65078-1-IG). Following overnight incubation, sections were washed three times in PBS for 10 min each, then incubated with appropriate secondary antibodies at 25 °C for 2 h. Nuclei were counterstained with DAPI (Beyotime) for 10 min. Finally, mounted sections were imaged using an A1 Si confocal microscope (Nikon), and images were analyzed using NIS-Elements software.

### TUNEL staining of brain sections

Apoptosis was assessed using TUNEL staining according to the manufacturer’s protocol (Elabscience). In brief, tissue sections were treated with proteinase K digestion for 10 min at 37 °C. The sections were then exposed to the TUNEL reaction mixture for 60 min at 37 °C in a dark, humidified chamber. Following nuclear counterstaining with DAPI (Beyotime), the apoptotic index was determined as the proportion of TUNEL-positive cells relative to total DAPI-labeled nuclei. ImageJ software (NIH) was employed for blinded quantitative assessment by an investigator unaware of experimental groupings.

### Western blot analysis

Following euthanasia, brain tissues from the peri-infarct region were rapidly collected through transcardiac perfusion with ice-cold PBS, with corresponding regions harvested from sham-operated mice as controls. The homogenates were sonicated (3 × 10 s pulses at 20% amplitude) to facilitate cell lysis and DNA shearing, thereby enhancing protein solubility and reducing viscosity. Lysates containing 30 µg protein were resolved via 12% SDS-PAGE and electrophoretically transferred to PVDF membranes. Membranes were immersed in blocking buffer (5% skim milk/TBST) for 2 h at ambient temperature prior to overnight incubation with primary antibodies at 4 °C: anti-FABP4 (1:200; Abcam; ab92501), anti-BACH1 (1:5,000; Proteintech; 14018-1-AP), anti-S100A9 (1:5000; Proteintech; 26992-1-AP), anti-Pecam1 (1:5,000; Abcam; ab222783), anti-Icam2 (1:2,000; Proteintech; 10121-2-AP), anti-Esam (1:2,000; Proteintech; 28739-1-AP), anti-Vcam1 (1:5,000; Proteintech; 83719-1-RR), and anti-β-actin (1:2,000; AC026; ABclonal). Subsequently, membranes were probed with HRP-conjugated species-specific secondary antibodies (1:5,000; anti-mouse or anti-rabbit; ABclonal) for 2 h at ambient temperature. After triple TBST rinses (5 min/wash), immunoreactive bands were detected using a ChemiDoc Imaging System (Bio-Rad, USA) and densitometrically analyzed with ImageJ. Quantitative data were normalized against β-actin and calculated as fold-changes versus sham-operated controls.

### Flow cytometry analysis

Cervical dislocation was employed to sacrifice mice. Then, mice underwent transcardiac perfusion with ice-cold PBS followed by rapid brain collection and immersion in chilled Hank’s Balanced Salt Solution (HBSS). Enzymatic digestion with 2 mg/ml Papain in HBSS was conducted at 37 °C for 1 h under gentle rotation. The resulting cell suspension was sequentially passed through a 50-µm nylon mesh and centrifuged at 300 × g for 5 min at 4 °C. For immunophenotyping, Cellular labeling was executed utilizing fluorescence-conjugated monoclonal antibodies: APC-Cy7 anti-mouse CD45 (Thermo, A15395), PE anti-mouse Ly6G (Thermo, 16–9668-82), Pacific Blue anti-mouse Ly6C (Biolegend, 128064), PE-Cy7 anti-mouse CD3e (Thermo, 25–0031-82), and APC anti-mouse CD8 (Thermo, 17–0081-82). Flow cytometry analysis was conducted using the NovoCyte 3000 flow cytometer, with data interpretation performed through NovoExpress Software (v1.4.1). To ensure analysis of viable single cells, we employed rigorous morphological gating based on FSC/SSC properties and pulse geometry (FSC-H vs. FSC-A), and maintained cells on ice throughout processing to minimize cell death.

### Liquid chromatography tandem mass spectrometry (LC-MS/MS) analysis for FABP4-interacting proteins

Protein samples obtained from FABP4 immunoprecipitation were analyzed by liquid chromatography-tandem mass spectrometry (LC-MS/MS), with IgG immunoprecipitation-derived samples serving as negative controls. Following silver staining verification on a 12% SDS-PAGE gel, excised protein bands were processed by PTM Biolabs, Inc. (Hangzhou, China) for proteomic analysis.

### Co-immunoprecipitation (Co-IP)

Cells were lysed in immunoprecipitation (IP) buffer (Thermo Scientific, 87787) supplemented with protease inhibitor cocktail (1:100) at 4 °C for 5 h with gentle rotation. Lysates were centrifuged at 12,000 × g for 15 min at 4 °C, and 1 mg of soluble protein supernatant was incubated with 4 µg of the following primary antibodies conjugated to protein A/G magnetic beads (ACE Biotechnology, BK0004-02): anti-ubiquitin (Proteintech, 10201-2-AP), anti-S100A9 (Proteintech, 26992-1-AP), and anti-FABP4 (Proteintech, 15872-1-AP) at 4 °C overnight (14 h) with end-over-end rotation. Antibody-antigen complexes were then captured by protein A/G magnetic beads at 4 °C.

### ChIP- qPCR assay

Cells were fixed using PFA (4%) before being lysed. Chromatin was subsequently isolated and subjected to enzymatic fragmentation. The dissociated chromatin underwent immunoprecipitation with either anti-BACH1 (Proteintech, 14018-1-AP) or IgG (Beyotime, A7016). Following immunoprecipitation, the protein-DNA complexes were separated and purified. The extracted DNA was subjected to agarose gel electrophoresis followed by quantitative PCR analysis.

### RNA-seq

Forty-eight hours after ICH, experimental subjects underwent terminal anesthesia prior to cerebral tissue harvesting using established protocols. Hematoma-adjacent regions (1–2 mm radius) were cryosectioned into 1-mm thick slices using precision surgical blades under cryogenic conditions (0–4 °C). Sham-operated controls underwent identical tissue procurement procedures excluding injury induction. After cooling the surrounding tissue in liquid nitrogen, we placed it in a −80 °C until library preparation.

### Quantitative real-time PCR (qRT-PCR)

Total RNA isolation was performed with TRIzol Reagent (Invitrogen, USA) in strict accordance with the standardized protocols. Quantitative analysis was carried out on a Bio-Rad Real-Time System. All primer sequences are cataloged in Supplementary Table S1, with β-actin serving as endogenous control and relative quantification performed via comparative Cq method (2 − ΔΔCq).

### Cell culture and treatment

Primary microglial cells were harvested from cerebral tissues of neonatal C57BL/6J mice through enzymatic dissociation according to established protocols. Following meningeal removal, brain tissue was mechanically dissociated and enzymatically treatment with 0.25% trypsin at 37 °C for 5 min, then passed through a 40-µm nylon mesh. The resulting cell suspension was resuspended in DMEM GlutaMAX medium containing 10% fetal bovine serum (Excell Bio) and 1% penicillin/streptomycin (Solarbio), followed by incubation in poly-D-lysine-precoated flasks for 7 days. Microglia were mechanically detached from mixed glial cultures and subsequently cultured in complete medium supplemented with 20 ng/ml macrophage colony-stimulating factor for an additional 7 days prior to experimentation. For primary neuron culture, C57BL/6 mice were euthanized at embryonic day 18. Cerebral cortices were dissected, minced into small fragments, and enzymatically dissociated with trypsin. Dissociated neurons were plated onto poly-L-ornithine/laminin (Sigma-Aldrich)-coated dishes and maintained in Neurobasal Medium (Gibco) supplemented with L-glutamine (Solarbio), B27 (Gibco), and penicillin/streptomycin (Beyotime) for 7 days. Plasmid and siRNA transfections were performed with Lipofectamine 2000 Reagent (Invitrogen, Carlsbad, California, USA) following the manufacturer’s protocol [61]. Specific gene primer sequences are listed in Table S1.

### Fabrication and functionalization of therapeutic MSN carriers

Following established protocols [62], the synthesis commenced by dissolving 2 g cetyltrimethylammonium chloride (CTAC) and 0.04 g triethanolamine (TEA) in 20 mL deionized water maintained at 95 °C under oil-bath conditions. After 1 h vigorous stirring, 1.5 mL tetraethyl orthosilicate (TEOS) was introduced dropwise for subsequent silica polycondensation over 60 min. The resulting MSNs underwent ethanol washing (3 cycles) post-centrifugation (27,500 ×g, 20 min), followed by template removal through 7 h calcination at 550 °C in a muffle furnace. For surface functionalization, 300 mg purified MSNs were sonicated in 30 mL methanol, then treated with 2 mL N-[3-(trimethoxysilyl)propyl]ethylenediamine for 24 h amine grafting. Amino-modified MSNs (MSN-NH₂) were isolated via ultracentrifugation (85,050 ×g, 10 min), methanol-rinsed, and vacuum-dried. Subsequent alkyne conjugation involved reacting 150 mg MSN-NH₂ with 1.5 mL propargyl bromide and 3 mL triethylamine in 30 mL methanol (24 h, ambient conditions). The alkyne-functionalized MSNs (MSN-Alkyne) were pelleted (5,020 ×g, 10 min), solvent-exchanged, and desiccated overnight. Therapeutic loading was achieved by incubating 1 mL FABP4 siRNA (100 µM) with 40 mL MSN-Alkyne suspension (1 mg/mL) under dark conditions (24 h). Post-centrifugation (5,628 ×g, 10 min) yielded siRNA-loaded carriers (MSN-siFABP4), which were then conjugated with 5 mg VCAM1-targeting peptide (VHPKQHRGC) via copper-catalyzed azide-alkyne cycloaddition [54]. Specifically, 30 mg MSN-siFABP4 and catalytic cocktail (1 mL containing 0.5 mM CuSO₄·5 H₂O + 1 mM sodium ascorbate) were reacted under nitrogen for 72 h to construct Vt-MSN-siFABP4 nanovectors.

### Analytical characterization

Morphological evaluation was performed using transmission electron microscopy (TEM, Tecnai G2 F30, FEI). Textural properties including surface area and pore distribution were determined through nitrogen physisorption measurements (ASAP 2460, Micromeritics). Surface charge profiles were acquired via electrophoretic light scattering (Zetasizer Nano-S90, Malvern Panalytical), while chemical functionalization was verified by FTIR spectroscopy (Nicolet Nexus, Thermo Scientific).

### Drug treatment and evaluation of brain targeting delivery

To assess cerebral targeting efficacy, mice were administered 200 µL of Vt-MSN-siFABP4 (comprising 2 mg/mL siFABP4 and 20 µg/mL Cy5.5) via daily intravenous injections. Following a 24-hour interval, in vivo fluorescence signals were quantified in real-time using the IVIS imaging platform. Subsequently, animals were humanely sacrificed for organ harvest (including brain, cardiac, pulmonary, hepatic, splenic, and renal tissues) to to conduct biodistribution analysis using the IVIS imaging system. For therapeutic intervention in ICH models, a daily intravenous regimen of 200 µL Vt-MSN-siFABP4 (2 mg/mL siFABP4) was systematically implemented, with the first dose administered at 4 h post-ICH induction.

### Statistical analysis

Results are expressed as mean ± SD unless indicated otherwise. Statistical significance between two groups was determined via bilateral unpaired t-tests. For three or more groups, one-way analysis of variance (ANOVA) followed by a Bonferroni post hoc test was applied. When analyzing multiple comparisons, two-way ANOVA with a Bonferroni post hoc test was utilized. Subjects were randomly allocated across experimental groups, with group sizes determined by calculations derived from prior mechanistic studies (detailed in figure legends). Mice exhibiting postoperative mortality were excluded from analysis. All experimental procedures, including group allocation, data acquisition, and statistical evaluation, were conducted under blinded conditions. Statistical significance was defined as *P* < 0.05. Analyses were performed using GraphPad Prism 9.5.

## Supplementary Information

Supplementary Material 1.

Supplementary Material 2.

## Data Availability

RNA-seq analysis on mouse brain has been deposited in Gene Expression Omnibus (GEO) under the accession number GSE295108. Experimental data related to these findings are accessible from the corresponding author upon justified request.
