# Supplementary material for: Fatty acid-binding protein 4 drives microglia-mediated neuroinflammation through promoting S100A9 expression and lipid droplet accumulation after intracerebral hemorrhage
Source: J Neuroinflammation. 2025 Nov 7;22:263. doi: 10.1186/s12974-025-03573-6 (PMC12595865; doi:10.1186/s12974-025-03573-6)
Supplement: Supplementary file 1 — Supplementary Material 1. [file 12974_2025_3573_MOESM1_ESM.docx]

**Supplementary Materials for**

**Fatty acid-binding protein 4 drives microglia-mediated neuroinflammation through promoting S100A9 expression and** **lipid droplet accumulation after intracerebral hemorrhage**

**The file includes:**

**Figs. S1 to S7**

**Table S1**

**
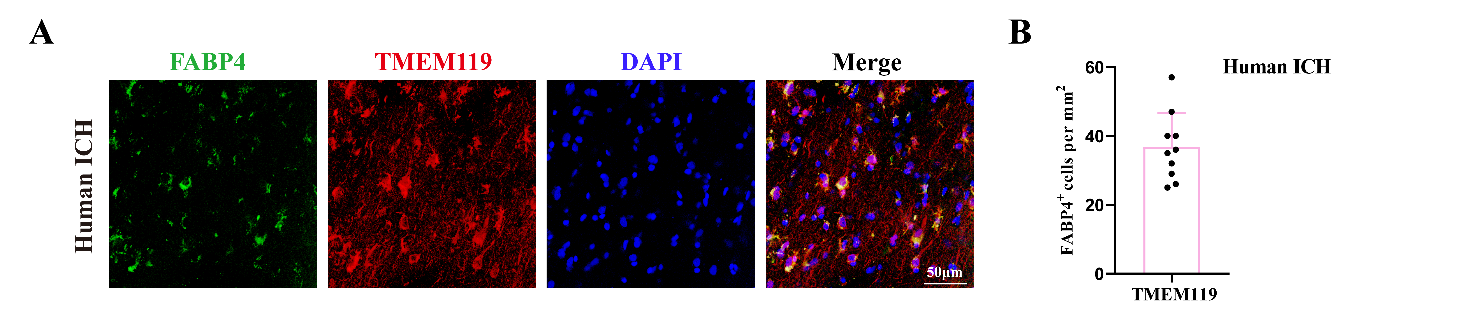
Supplementary Figures**

**Figure S1. FABP4 exhibits specific expression in microglial cells. (A-B)** Representative immunostaining images of FABP4 expression in microglia (TMEM119⁺ cells) in brain tissue from ICH patients. Scale bar: 50 μm. n = 10.


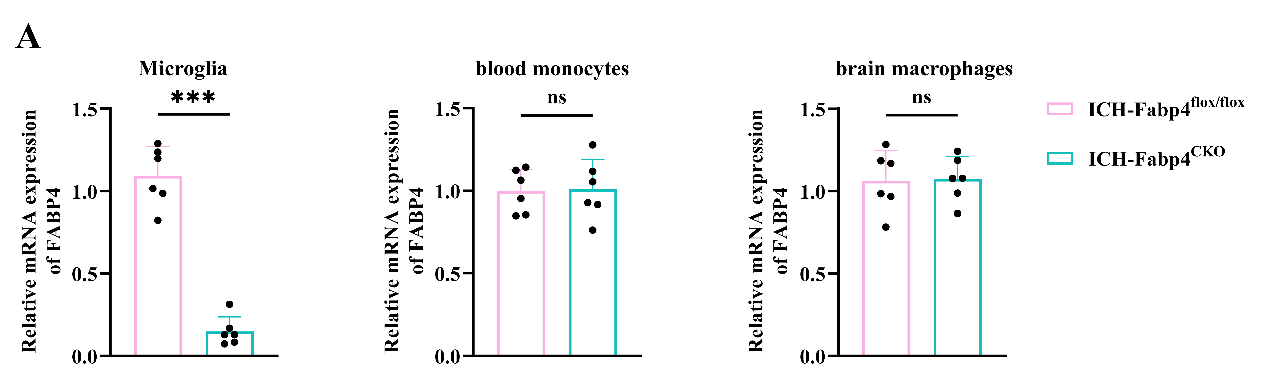


**Figure S2.** **The knockout of Fabp4 is specific to microglia. (A)** qPCR was used to analyze FABP4 levels in different cell types across the experimental groups (n = 6 per group, Student’s t-test). Data are presented as means ± SD. *p < 0.05, **p < 0.01, ***p < 0.001.


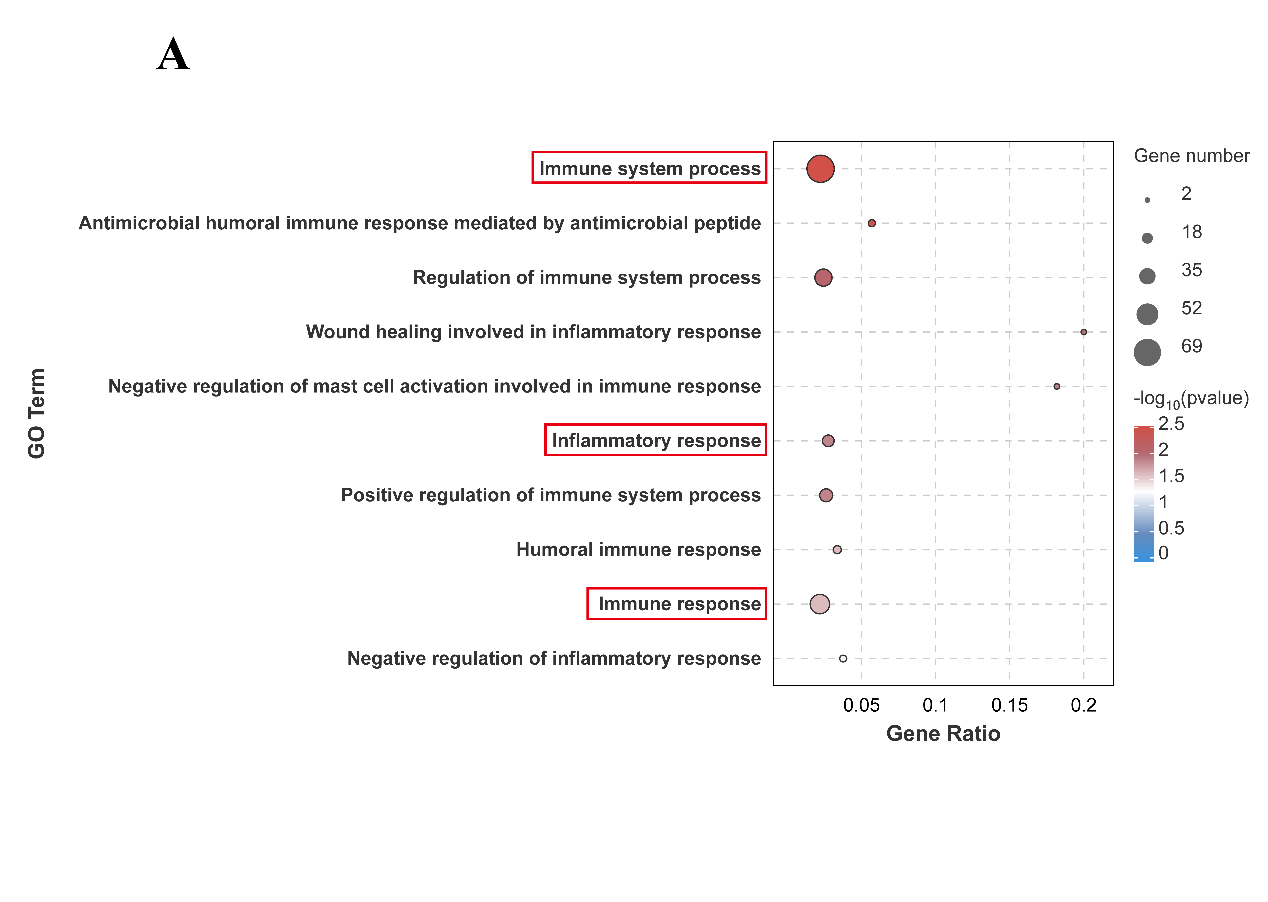


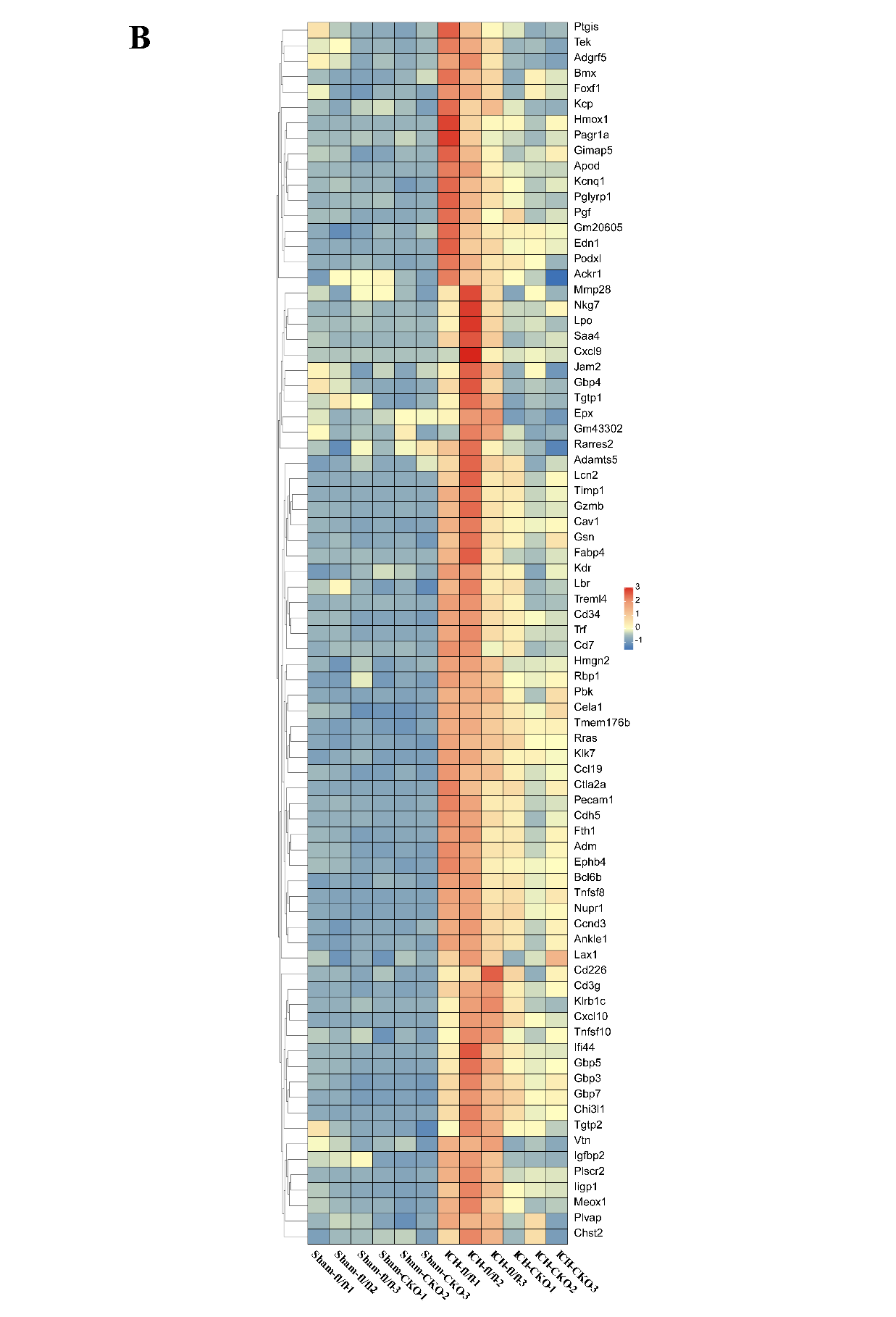


**Figure S3. RNA sequencing revealed that Fabp4 knockout reversed the expression of the majority of inflammatory genes. (A)** GO enrichment analysis of the 345 genes regulated by FABP4. **(B)** Heat map showed the changes in the expression of inflammation-related genes in each group.


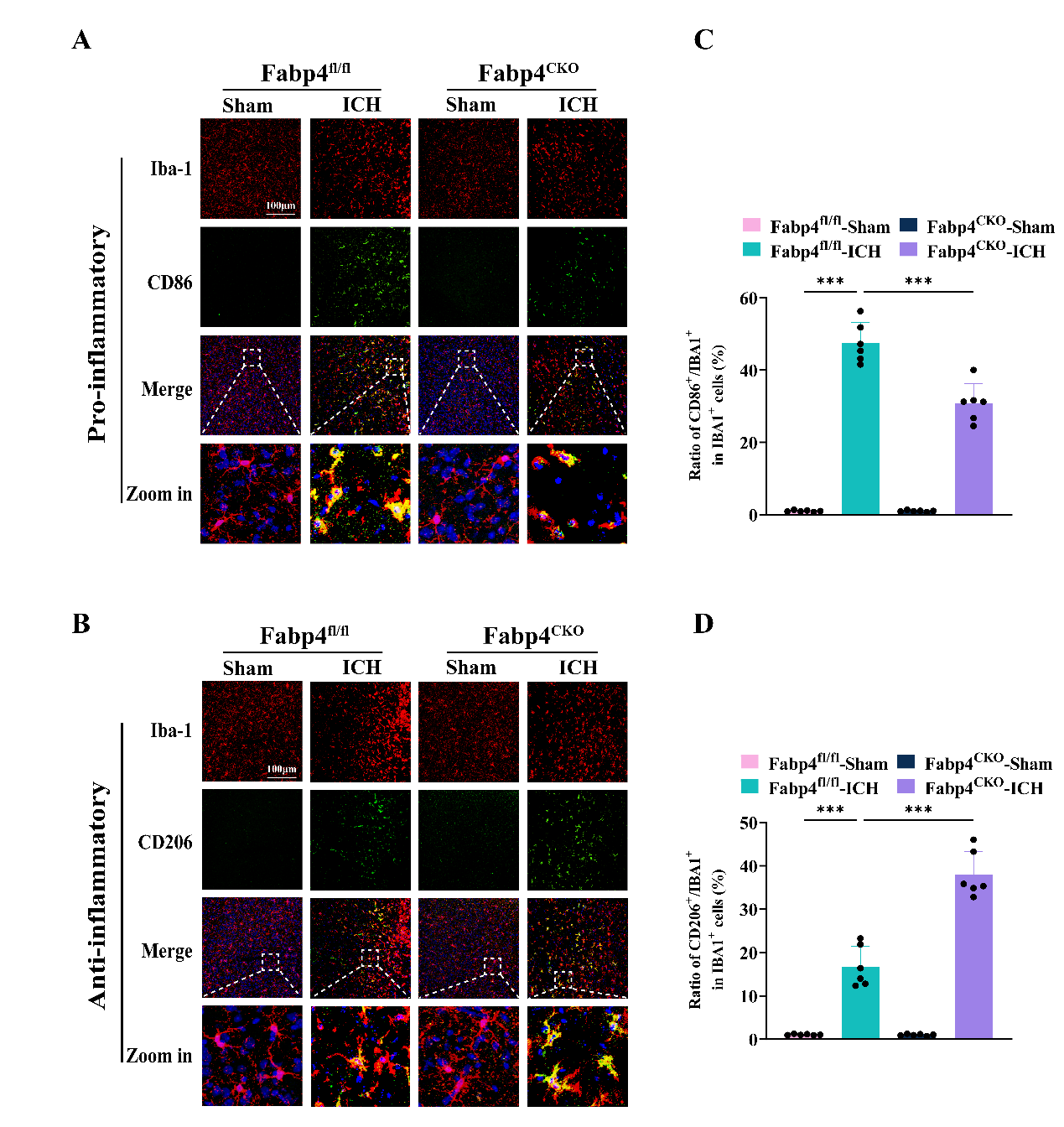


**Figure S4.** **Effects of Fabp4 knockout on microglia polarization at 48h after ICH. (A**-**B)** Representative images of immunofluorescence staining of CD86 (green) and CD206 (green) with Iba-1 (red) in the perihematomal area at 48h after ICH. Scale bar: 100 μm. **(C**-**D)** Quantification of CD86⁺/IBA1⁺ and CD206⁺/IBA1⁺ ratios in microglia (%) (n = 6 per group, one-way ANOVA). Data are presented as means ± SD. *p < 0.05, **p < 0.01, ***p < 0.001.

**
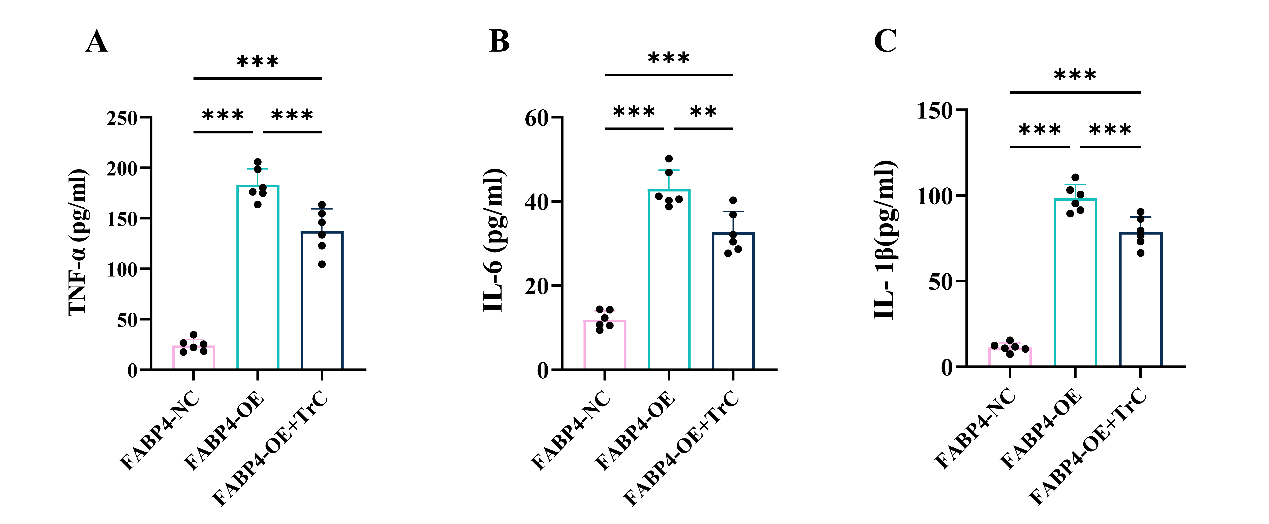
**

**
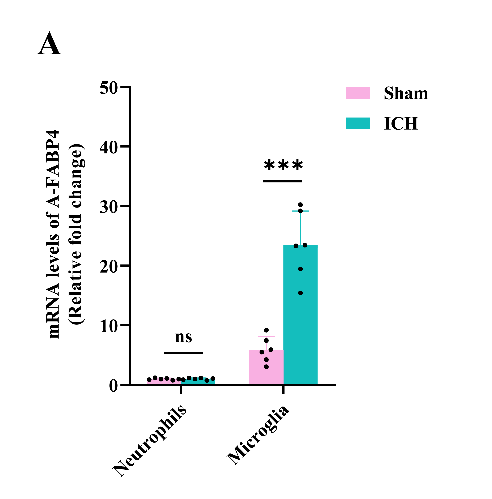
Figure S5.** **Pharmacological inhibition of lipid droplet biogenesis in primary microglia attenuated FABP4 overexpression-induced pro-inflammatory activation. (A**-**C)** Quantitative measurement of pro-inflammatory factors (TNF-𝛼, IL-6, and IL-1𝛽) in FABP4-OE primary microglia treated with Trc using ELISA (n = 6 per group, one-way ANOVA). Data are presented as means ± SD. *p < 0.05, **p < 0.01, ***p < 0.001. **Figure S6. FABP4 expression was significantly upregulated in microglia but remained unchanged in neutrophils after ICH. (A)** Relative expression of FABP4 in neutrophils and microglia as determined by qPCR (n = 6 per group, Student’s t-test). Data are presented as means ± SD. *p < 0.05, **p < 0.01, ***p < 0.001.

**
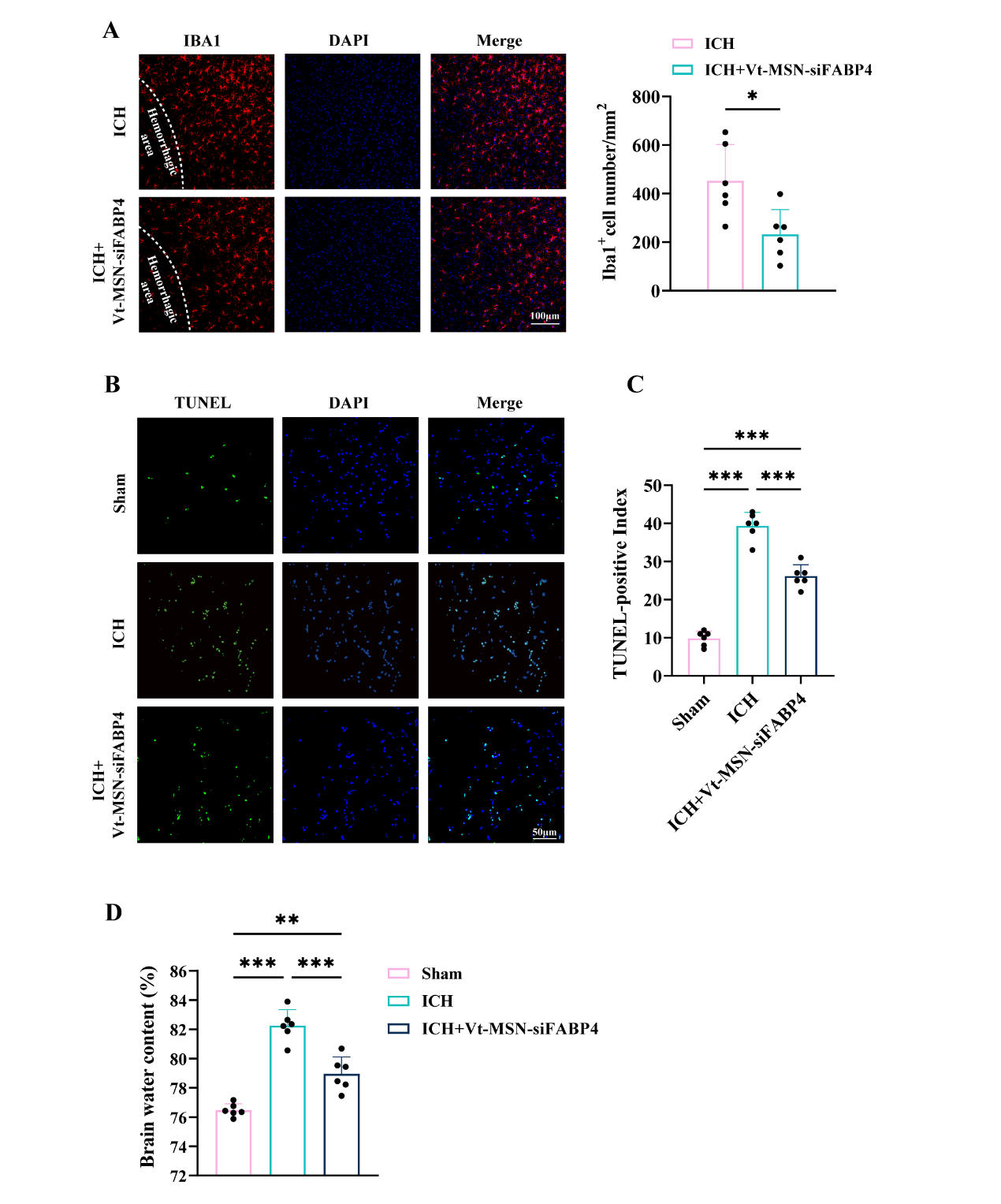
Figure S7.** **VCAM-1 targeted MSN delivery of siFABP4 attenuated microglial activation and decreased the TUNEL-positive cell ratio and brain edema in ICH mice (A)** Evaluation of immunoreactivity for the microglial marker Iba1 and quantification of activated microglia were performed under different treatment conditions at day 2 after ICH. Scale bar: 100 μm. Dotted line denotes hemorrhagic border (n = 6 per group, Student’s t-test). **(B-C)** Brain sections of mice showed representative images and statistical analysis of TUNEL-positive cells 48 hours after ICH in each group (n = 6 per group, one-way ANOVA). Scale bar: 50 μm. **(D)** Quantification of brain water content at 48 h after ICH (n = 6 per group, one-way ANOVA). Data are presented as means ± SD. *p < 0.05, **p < 0.01, ***p < 0.001.

**Table S1**

| Protein Name | Forward Primer） | Reverse Primer (5'-3'） |
| --- | --- | --- |
| FABP4 | 5'-GGGGCCAGGCTTCTATTCC-3' | 5'-GGAGCTGGGTTAGGTATGGG-3' |
| BACH1 | 5'-GAACAG GGCTAC TCGCAAAG-3' | 5'-AAAGGGCAGTTGACGGAA C-3' |

| siRNA | Sequence |
| --- | --- |
| Control siRNA | 5'-UUAUGCCGAUCGCGUCACATT-3'  3'-TTAAUACGGCUAGCGCAGUGU-5' |
| FABP4 siRNA | 5'-GACGUUGACCUGGACUGAAdTdT-3'  3'-UUCAGUCCAGGUCAACGUCdTdT-5' |
| BACH1 siRNA control | Empty Vector Control |
| BACH1 siRNA | F:5'-GCGTACACAATATCGAGGATTCAAGACGTC  CTCGATATTGTGT ACGCTTTTTT-3'  R:5'-AAAAAAGCGTACACAATATCGAGGACGTCT  TGAATCCTCGATA TTGTGTACGC-3' |
