# Supplementary material for: Fatty acid-binding protein 4 drives microglia-mediated neuroinflammation through promoting S100A9 expression and lipid droplet accumulation after intracerebral hemorrhage
Source: J Neuroinflammation. 2025 Nov 7;22:263. doi: 10.1186/s12974-025-03573-6 (PMC12595865; doi:10.1186/s12974-025-03573-6)

Full unedited gel for Figure 1 A

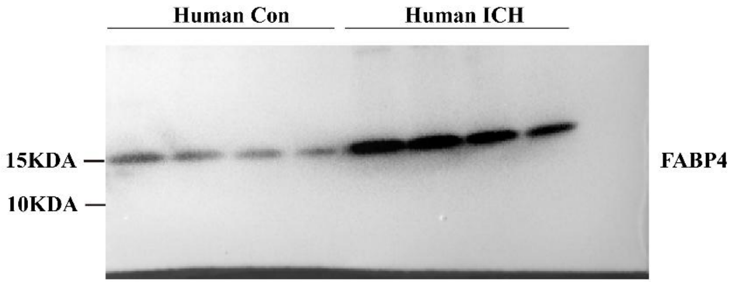

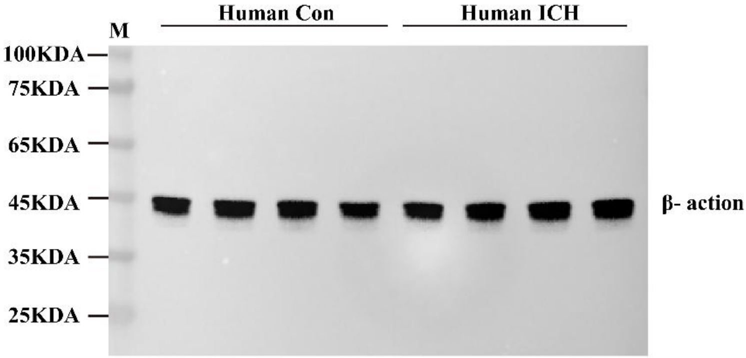

Full unedited gel for Figure 2 A

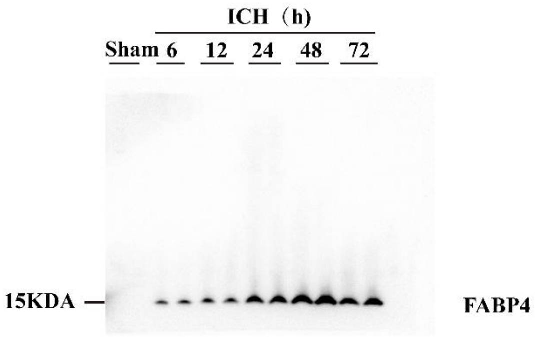

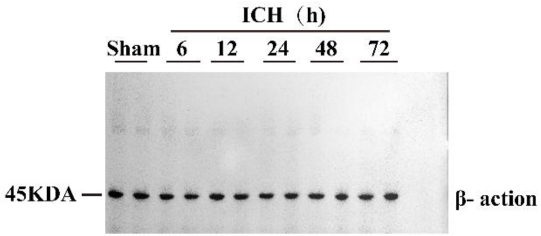

Full unedited gel for Figure 2 J

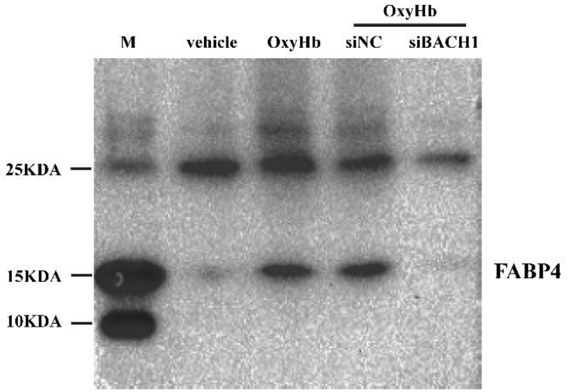

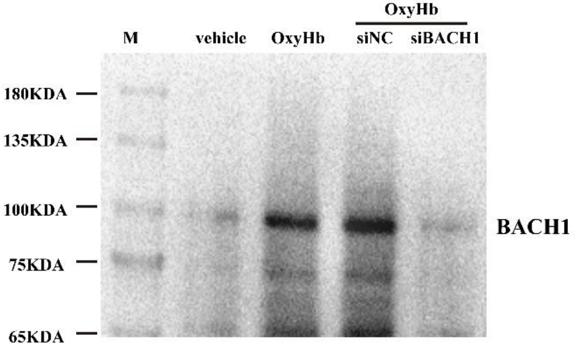

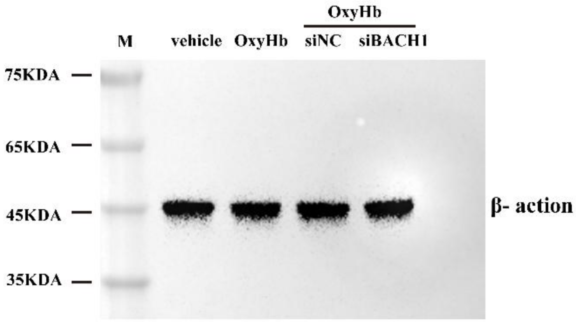

Full unedited gel for Figure 5 C

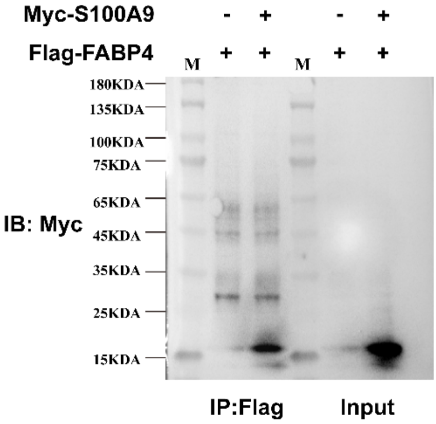

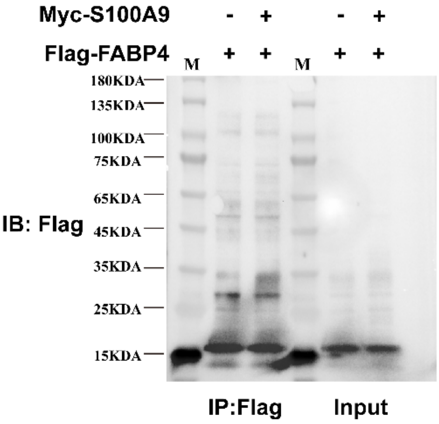

Full unedited gel for Figure 5 D

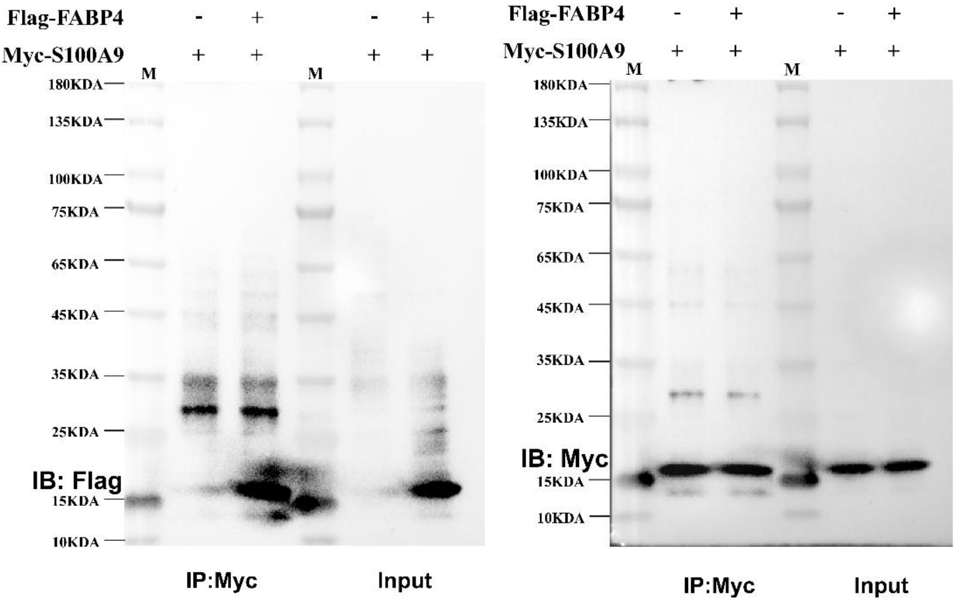

Full unedited gel for Figure 5 E

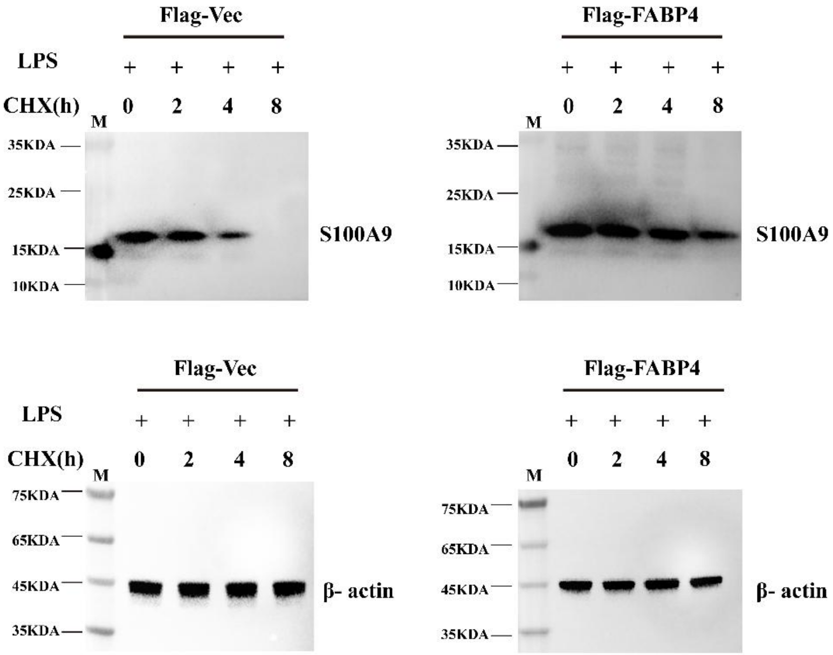

Full unedited gel for Figure 5 F

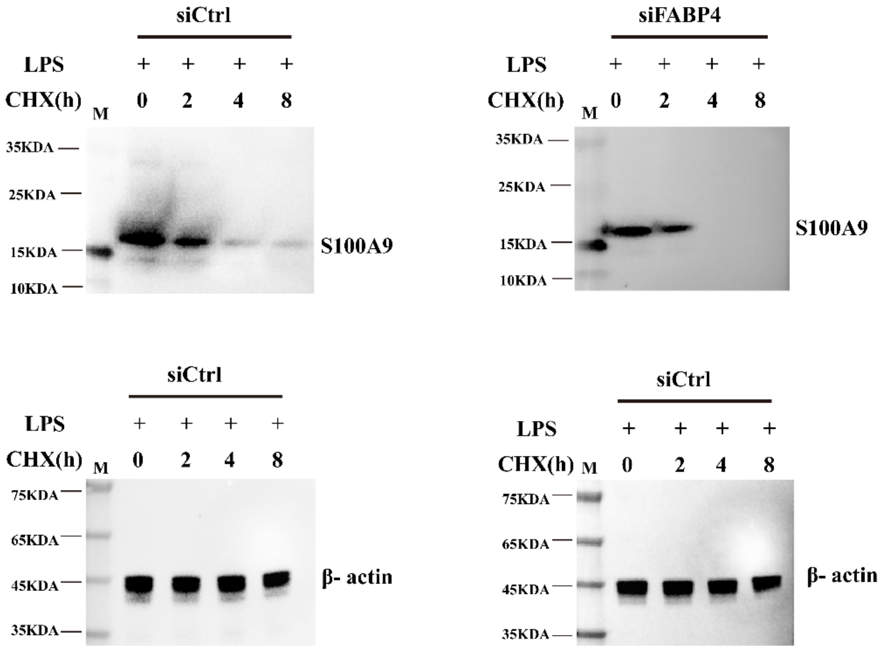

Full unedited gel for Figure 5 I

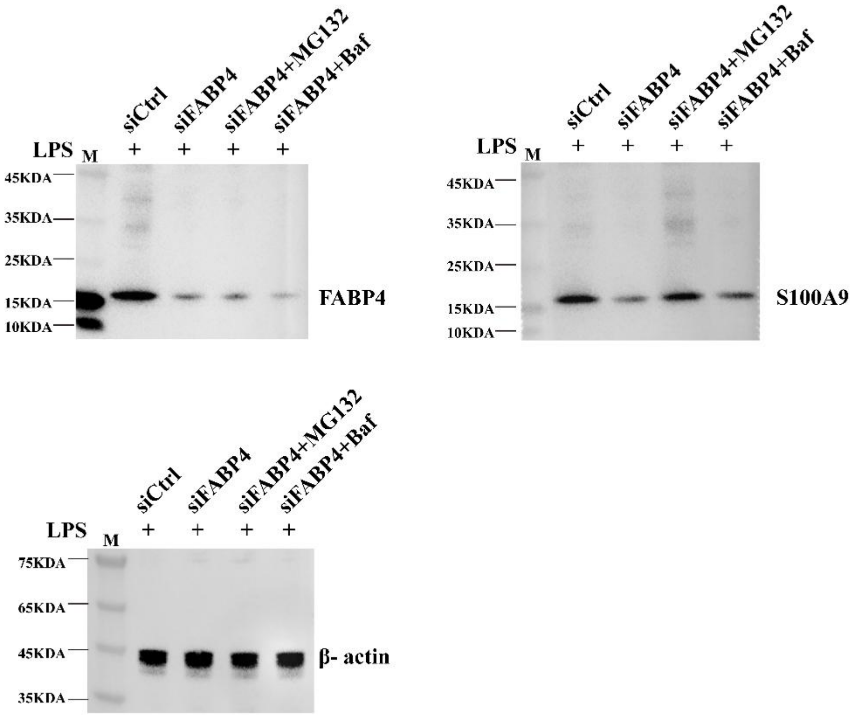

Full unedited gel for Figure 5 J

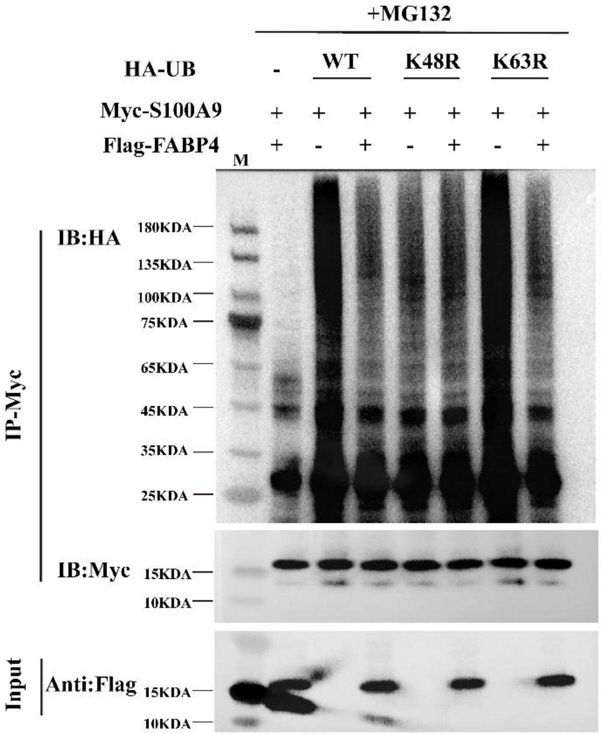

Full unedited gel for Figure 6 C

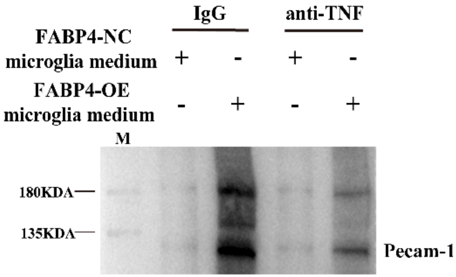

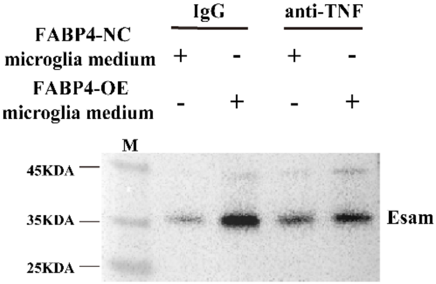

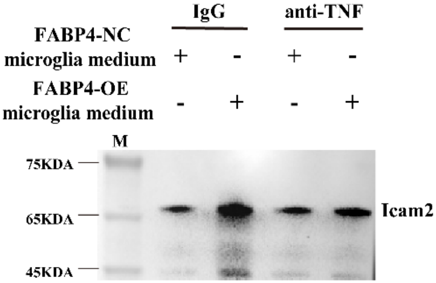

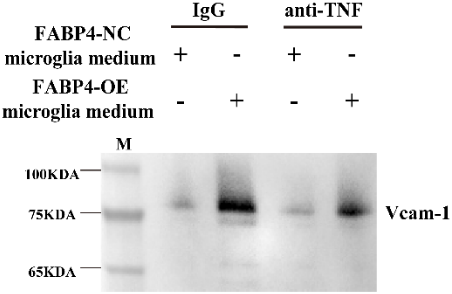

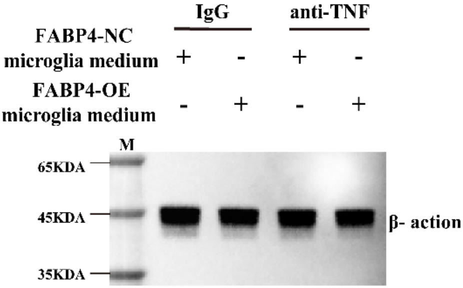

Full unedited gel for Figure 7 F

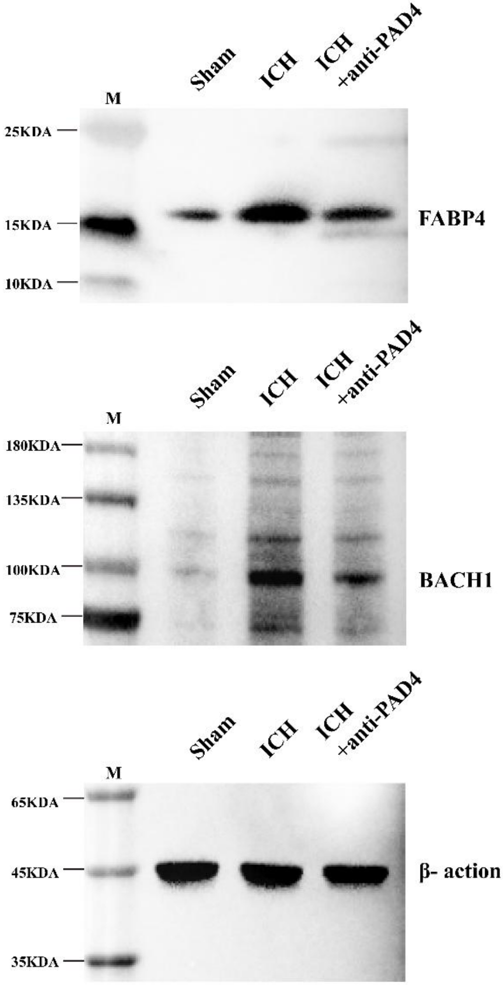

Supplement: Supplementary file 2 — Supplementary Material 2. [file 12974_2025_3573_MOESM2_ESM.pdf]
